# Revisiting the evolution of bow-tie architecture in signaling networks

**DOI:** 10.1038/s41540-024-00396-8

**Published:** 2024-06-29

**Authors:** Thoma Itoh, Yohei Kondo, Kazuhiro Aoki, Nen Saito

**Affiliations:** 1 National Institute for Basic Biology, National Institutes of Natural Sciences, 5-1 Higashiyama, Myodaiji-cho, Okazaki, Aichi 444-8787 Japan; 2 Department of Basic Biology, School of Life Science, SOKENDAI (The Graduate University for Advanced Studies), 5-1 Higashiyama, Myodaiji-cho, Okazaki, Aichi 444-8787 Japan; 3 Exploratory Research Center on Life and Living Systems (ExCELLS), National Institutes of Natural Sciences, 5-1 Higashiyama, Myodaiji-cho, Okazaki, Aichi 444-8787 Japan; 4 Graduate School of Integrated Sciences for Life, Hiroshima University, Higashihiroshima, Hiroshima 739-8511 Japan

**Keywords:** Biochemical networks, Evolvability

## Abstract

Bow-tie architecture is a layered network structure that has a narrow middle layer with multiple inputs and outputs. Such structures are widely seen in the molecular networks in cells, suggesting that a universal evolutionary mechanism underlies the emergence of bow-tie architecture. The previous theoretical studies have implemented evolutionary simulations of the feedforward network to satisfy a given input-output goal and proposed that the bow-tie architecture emerges when the ideal input-output relation is given as a rank-deficient matrix with mutations in network link intensities in a multiplicative manner. Here, we report that the bow-tie network inevitably appears when the link intensities representing molecular interactions are small at the initial condition of the evolutionary simulation, regardless of the rank of the goal matrix. Our dynamical system analysis clarifies the mechanisms underlying the emergence of the bow-tie structure. Further, we demonstrate that the increase in the input-output matrix reduces the width of the middle layer, resulting in the emergence of bow-tie architecture, even when evolution starts from large link intensities. Our data suggest that bow-tie architecture emerges as a side effect of evolution rather than as a result of evolutionary adaptation.

## Introduction

**Fig. 1:**
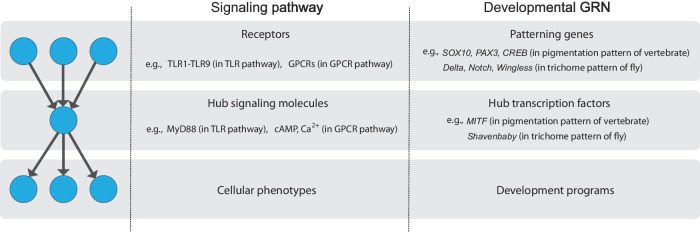
Universality of bow-tie architecture in a biological network. Bow-tie architecture is a hierarchical network having a narrow middle layer. A schematic of the typical bow-tie architecture is shown in the far-left column. The circles (nodes) represent molecules and the arrows represent reactions or information flows. The other columns provide examples of bow-tie architecture in a signaling network and a developmental gene regulatory network.

Many signaling and gene regulatory networks have hitherto been reported to exhibit bow-tie architecture, also known as an hourglass structure^1–3^, which employs material or informational flows and is characterized by hierarchical network structures with a narrow middle layer and multiple inputs and outputs. The network with bow-tie architecture receives various inputs and then converges signals into a few middle layer components known as waists, cores, or knots. Subsequently, the waist components regulate a wide range of downstream outputs^1,4^. Another definition of bow-tie architecture has been proposed and used mainly in the context of the metabolic network^5–8^ in this definition, the bow-tie architecture is defined as a network that has few input/output nodes and one giant middle module consisting of many interconnected components. However, we will not discuss this type of bow-tie architecture in this study. Bow-tie architecture has been widely found in signaling networks^9–16^ (Fig. 1). For example, in the toll-like receptor (TLR) signaling network, various TLRs recognize pathogen-associated molecular patterns and the signals are mediated through MyD88, resulting in the expression of various immune response genes^14,16^. In the G-protein coupled receptor (GPCR) signaling network, a vast number of GPCRs encoded in the human genome primarily activate a few G proteins followed by the regulation of the calcium ion or cAMP concentration, which nonetheless lead to the induction of various gene expressions causing diverse cellular phenotypes^17–19^. Bow-tie architecture is also found in the gene regulatory network. For example, in the gene regulatory network involved in the body development, information from development patterning genes is integrated into the cis-regulatory region of the hub transcription factors, which regulates a large number of developmental programs^20–22^. As distinct from these networks of information flow, bow-tie architecture is also found in the metabolic network that supports energy and material flow^3,23^.

Bow-tie architecture has also been reported in non-biological networks, such as internet protocols^24,25^ railroad transportation systems^4^, and so on. This universality of the bow-tie architecture implies the existence of design principles underlying these systems, and thus it is important to elucidate how and why the bow-tie architecture emerges.

Despite the ubiquity of bow-tie architecture in living systems, the driving forces behind its emergence are still being debated. Kitano et al. have argued that bow-tie architecture is the result of optimization in the trade-off among robustness, fragility, resource limitation, and performance^1^. Polouliakh et al. and Yan et al. have shown that a narrow intermediate layer in the bow-tie architecture provides the capability to classify the inputs^17,26^. In terms of control theory, Wang et al. have proposed that bow-tie architecture possesses controllability^27^, and in line with this idea, Kitano and Ni et al. have suggested that bow-tie architecture retains high evolvability, with a capacity for adjusting the outputs to the environment^1,28^. Although these studies have shown the potential functions or advantages of bow-tie architecture, how this architecture evolved has not yet been elucidated.

**Fig. 2:**
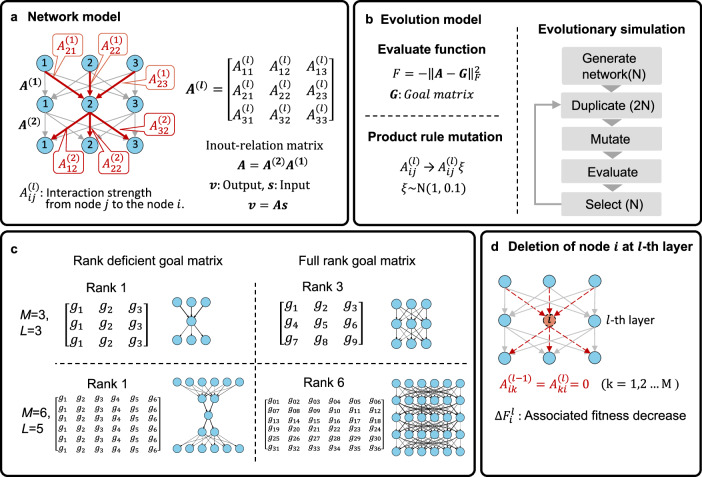
Evolutionary simulation of bow-tie architecture. **a** A linear network model. The network is modeled as a hierarchical linear network. All links between layers are described by a matrix. The ${ij}$ element, ${A}_{{ij}}^{(l)}$, represents the output strength from node $j$ in layer $l$ to node $i$ in layer $l$ + 1. The product of these matrices produces the in-out matrix of network $A$. **b** Evolutionary model of the network. The evaluate function is defined by the distance between in-out matrix $\bf A$ and goal matrix ${\bf G}.$ $\|\cdot \|_{F}^{2}$ is the square of the Frobenius norm. Mutation is modeled as the product of link intensity ${A}_{{ij}}^{(l)}$ and ${\rm{\xi }}$ generated from a Gaussian distribution. Evolution is simulated by repeating duplication, mutation, evaluation, and selection. **c** A different rank of the goal matrix is randomly generated. The typical evolved network under the given goal matrix is shown next to the goal matrix. Bow-tie architecture emerges under the rank-deficient goal matrix in the end state. **d** Deletion of node $i$ at the $l$ th layer. The effect of node $i$ is evaluated by assessing the fitness decrease upon removal of node $i$. Removal of node $i$ is executed by ${A}_{{ik}}^{(l-1)}={A}_{{ki}}^{(l)}$ = 0.

Friedlander et al. proposed an evolutionary mechanism of bow-tie architecture based on their simulation using a linear network model^2^, in which the biological networks, specifically signaling networks, were modeled by a set of matrices describing the network structure (Fig. 2a). They demonstrated that the bow-tie architecture emerges when (1) the mutations occur in a multiplicative manner, and (2) the network evolves toward a certain target in-out relation (i.e., the goal matrix; see Fig. 2c) that is expressed by a rank-deficient matrix. The condition (1) models the characteristics of biological mutations in the signaling network or gene regulatory network, which have been reported to be multiplicative^29,30^ and tend to minimize link intensity^31^. The condition (2) reflects biological inputs that are often redundant^32^, such as image inputs in the retinal neural network^31^ or TLR signaling pathways^33^. Although this model assumes linear interaction for simplification, recent studies reported that the linearity is observed at the level of the circuit in chemotactic signaling systems in animal networks^34^, suggesting that linear interaction can capture qualitative behaviors and is not an inappropriate abstraction.

A previous study^2^ proposed a clear formulation for addressing the bow-tie evolution and presented a plausible hypothesis. However, its evolutionary dynamics remain unclear. In this study, we investigate a linear network model as a model of the signaling network, and report that bow-tie inevitably emerges regardless of goal matrix rank in the early phase of evolution when link intensities representing molecular interactions are small in the initial conditions of evolutionary simulations. This indicates that the network structure at the beginning of the evolution plays a crucial role in the emergence of bow-tie architecture. Furthermore, we analyzed the mechanism of the emergence of bow-tie architecture by using a simple ODE model. Based on the identified mechanism of the emergence of bow-tie architecture, we also suggest that environmental fluctuation or an increase in the number of input/output nodes, which is naturally considered in the biological evolution, facilitates the emergence of bow-tie architecture. Our study addresses the bow-tie architectures in which information flow is transmitted through a few intermediate nodes, such as the architectures found in signal transduction and gene regulatory networks. Although bow-tie structure in metabolic networks is also important, we exclude it from the scope of this study as the network of material flows with conservation laws makes the problem much more difficult.

### Model

To investigate the evolution of the bow-tie architecture, we consider layered feedforward networks with *M* nodes in each layer. We adopted the linear network model proposed by ref. ^2^, in which link intensities from the *l*th layer to the *l* + 1 th layer are described by the *M* × *M* matrix ${\bf A}^{{\boldsymbol{(}}l{\boldsymbol{)}}}$ (Fig. 2a). The ${ij}$ element, ${A}_{{ij}}^{(l)}$, is the link intensity from node *j* in the *l*th layer to node *i* in the *l* + 1 th layer and is defined as ${A}_{{ij}}^{(l)} \,>\, 0$. When an input signal ***s*** is applied to the network with *L* + 1 layers, output vector ***v*** is obtained as the product of matrices ${\bf{As}}\,{\boldsymbol{=}}\,{\bf{v}}$, where ${\bf{A}}$ is the in-out relation matrix of the network defined by ${{\bf{A}}\,=\,{\bf{A}}}^{(L)}{{\bf{A}}}^{(L-1)}...{{\bf{A}}}^{(1)}$ (Fig. 2a). A detailed explanation of the linear network model is given in Supplementary Fig. 1. The network evolves toward an ideal in-out relation matrix ***G*** (hereinafter referred to as the goal matrix) (Fig. 2b). We adopted a randomly generated goal matrix with different ranks (Fig. 2c). The evaluation function, i.e., fitness, is defined by$\,F=-\|{\bf{A}}-{\bf{G}}\|_{F}^{2}$, where the square of the Frobenius norm $\|\cdot \|_{F}^{2}$ represents the sum of the square of all matrix elements, and thus -$F$ denotes the distance between the in-out relation matrix ***A*** and the goal matrix ***G***. We evolved the network by optimizing the in-out relation matrix ***A*** so that fitness $F$ is maximized through the following genetic algorithm (Fig. 2b). We generate *N* individuals having an *L* + 1 layered network represented by a set of matrices ${{\bf{A}}}^{(1)},\ldots ,{{\bf{A}}}^{(L)}$. In each generation during evolution, the individuals are duplicated so that the population size is 2 *N* and mutations are randomly introduced into 20% of individuals in that population. For the mutations, following Friedlander et al., we adopted the product rule mutation^2^ and a mutation rate of 0.2 per population, which is the same as the ref.^2^. A randomly selected network link ${A}_{{ij}}^{(l)}$ is altered as ${A}_{{ij}}^{(l)}$→${A}_{{ij}}^{(l)}\xi$ with a random number $\xi$ generated from $N(1,\,0.1)$, i.e., a Gaussian distribution with mean one and variance 0.1. Variance 0.1 is selected to guarantee that $\xi$ does not take a negative value but has finite variance. Then, the fitness values *F* for 2 *N* individuals are evaluated, and finally the top *N* individuals are selected by tournament selection with group size 4^2,31,35^. Evolution is simulated by repeating these processes (Fig. 2b). The individuals are considered to be fully evolved when their average fitness value reaches −0.01 or higher, and the network of individuals with the highest fitness ${F}$ is analyzed. Throughout this study, the population size *N* = 100 is used. Whether network architecture is bow-tie or not is judged by calculating the decrease in fitness associated with the removal of each node. Deletion of node $i$ at the *l*th layer is implemented by setting ${A}_{{ik}}^{(l-1)}={A}_{{ki}}^{(l)}=0\,(k=\mathrm{1,2}\ldots M\,$), and the absolute value of the associated fitness decrease is denoted by $\Delta {F}_{i}^{l}$ (Fig. 2d). The ratio of fitness decrease ${P}_{i}^{l}$ within *l*th layer is then estimated as ${P}_{i}^{l}=\frac{\Delta {F}_{i}^{l}}{{\sum }_{i}^{M}\Delta {F}_{i}^{l}}$. We define an active node as a node whose removal causes a decrease in fitness satisfying a criterion ${P}_{i}^{l}\, >$ 0.001. When the number of the active nodes in a middle layer (i.e., the layer from *l* = 2 to *L* − 1) is smaller than *M*, the network is defined as a bow-tie network.

## Results

### Bow-tie transiently emerges regardless of goal when initial link intensities are small

**Fig. 3:**
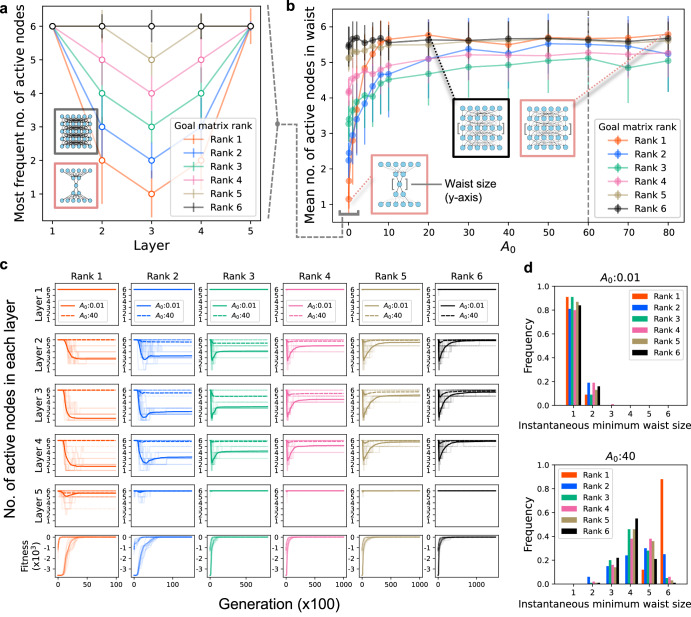
Simulation results with a network of 6 nodes x 5 layers. **a**, **b** Statistics of the evolved networks. **a** Reproduction of the previous results^2^. The Y-axis shows the mode among 100 runs of the number of active nodes in the most-adapted network. The initial link intensity is ${A}_{0}:0.01$. The error bars represent the standard deviation. Goal matrix elements are randomized under the conditions of rank 1 (red), rank 2 (blue), rank 3 (green), rank 4 (pink), rank 5 (brown) and rank 6 (black). The norm $\|{\bf{G}}\|_{F}$ is normalized to the same value ($\|{\bf{G}}\|_{F}$ = 60). The typical structures of the evolved network are shown within boxes. **b** The bow-tie emergence depends on the initial link intensity. X-axis: Link intensity of the initial network. Y-axis: Mean number of active nodes in the waist layer in an adapted network among 100 runs. The error bars are the standard deviation. The dashed line shows $\|{\bf{G}}\|_{F}=60$. Network structures in the box are the typical evolved network in the indicated point. The point enclosed by the bracket (${A}_{0}:0.01$) corresponds to Fig. 1a. **c**, **d** Statistics during evolution. **c** Evolution trajectories of the number of nodes in each layer. The bottom panel shows the average fitness trajectory. Simulation starts from ${A}_{0}=0.01$ in the solid lines and ${A}_{0}=40$ in the dashed lines. Trajectories are averaged among independent simulation runs (*n* = *100* for each color). The simulation runs that reach *F* > −0.01 are used. **d** Distribution of the instantaneous minimum waist width that the network experienced during evolution (*n* = 100 for each color).

First, we attempted to reproduce the simulation of ref. ^2^. by using network of 6 nodes × 5 layers, i.e., *M* = 6 and *L* = 4 (Fig. 3a). We started the evolutionary simulation from a random configuration of ${A}_{{ik}}^{(l)}$ with a small initial link intensity ${A}_{0}=0.01$, which is defined by the Frobenius norm of the initial in-out relation matrix $\|{\bf{A}}\|_{F}$. We were able to reproduce the previous results^2^, confirming that the network evolves to the bow-tie architecture when the goal matrix is rank-deficient (Fig. 3a). Although we adopted tournament selection following the previous study^2^, we also confirmed that almost the same result was obtained by adopting elite selection (Supplementary Fig. 2). Figure 3a illustrates the most frequent number of active nodes for each layer in the evolved network. We here refer to a middle layer with the smallest number of active nodes as a “waist” and denote its number of active nodes as the “waist size”. The waist size decreases as the rank of the goal matrix decreases; for a low-rank goal matrix, the network with a narrow waist size evolves, while a network with a waist size = *M* appears for the full-rank goal matrix (Fig. 3).

We simulated the evolution of a network starting from various values of ${A}_{0}$. Figure 3b shows the waist size of the evolved network against ${A}_{0}$, in which each dot represents the average value of over 100 runs, and illustrates that the bow-tie architecture evolves only at a range of ${A}_{0}$ (0.1–10). This range is quite narrow compared with the Frobenius norm of the goal matrix, i.e., 60, denoted by the black dashed line in Fig. 3b. When we started the simulation with an ${A}_{0}$ larger than 10, the network did not converge to a bow-tie structure in most cases (specifically for rank = 1). The probabilities for the bow-tie network with waist size = 1 exhibit a sharp transition around ${A}_{0}\, \sim \,2.5$ as ${A}_{0}$ decreases, while the probabilities for the bow-tie with waist size <5 exhibit a transition around ${A}_{0}$~ 10 in rank 1 and gradual decreases in the ranks 2 and 3 (Supplementary Fig. 3a).

The evolutionary dynamics also exhibited a remarkable dependence on initial link intensity ${A}_{0}$. Figure 3c shows the time series of the number of active nodes in each layer for a large (=40) and small (=0.01) ${A}_{0}$ with different goal ranks. When the goal matrix was full-ranked, the number of the active nodes in the 2–4th layers transiently decreased and then converged to 6 for the small ${A}_{0}$ (Fig. 3c; rank 6, solid black line), indicating a transient emergence of the bow-tie architecture. For the large ${A}_{0}$, the number of active nodes did not show such a transient drop (Fig. 3c; rank 6, dashed black line). When the goal rank was low, the network starting from the small ${A}_{0}$ converged to the bow-tie network (Fig. 3c; ranks 1–5, solid line), while the network starting from the large ${A}_{0}$ did not show even a transient emergence of the bow-tie architecture (Fig. 3c; ranks 1–5, dashed line). Although the network shows different evolutionary trajectories that depend on the goal rank, the transient decrease of waist size in the early phase of evolution can be observed in all ranks when evolution starts from the small ${A}_{0}$ (Fig. 3c and Supplementary Fig. 3b). Figure 3c elucidates that the waist width takes a minimum value during a rapid increase in fitness (the bottom panels) and this transient bow-tie structure is maintained even after the fitness is almost saturated. This transient emergence of bow-tie architecture was also confirmed for a larger network with 12 nodes × 5 layers (Supplementary Fig. 4) and for the other mutation rate (Supplementary Fig. 5).

Figure 3d shows the distribution of the instantaneous minimum waist size, which means the narrowest waist widths that the network reached during the course of evolution, and indicates that the network transiently evolves to bow-tie architecture regardless of the rank of the goal matrix if it starts from a small ${A}_{0}$ (Fig. 3d; upper panel), while bow-tie architecture could not be produced even in the low-rank goal matrix when the network started from a large ${A}_{0}$ (Fig. 3d; lower panel). The instantaneous minimum waist size as a function of ${A}_{0}$ shows bow-tie emergence in the range of ${A}_{0} \sim 10$ in all ranks (Supplementary Fig. 3b). The transient emergence of bow-tie, and its dependence on the ${A}_{0}$, are also observed by using the alternative active node definitions (i.e., the relative contribution of a node to the total in-out relation and relative value of the maximum interaction associated with a node; see Methods and Supplementary Fig. 6). These results imply that bow-tie architecture can spontaneously emerge without low rank goal. The low-rank goal just determines whether or not the transiently emerged bow-tie architecture is maintained.

Furthermore, we also found that the bow-tie emergence weakly depends on the variance of goal matrix elements, as well as the initial value ${A}_{0}$. Since the rank 1 goal has fewer independent elements than the rank 6 goal, the variance of the rank 1 goal tends to be smaller than that of the rank 6 goal. To eliminate this bias, we normalized the variance and norm (see Methods and Supplementary Fig. 7). With this normalized goal, the condition of ${A}_{0}$ for the bow-tie emergence is more apparent as ${0\, <\, A}_{0}\,\le\, 10$ (Supplementary Fig. 7a). Consistent with this finding, the probability for a bow-tie network with waist size <5 increases with decreasing *A*_0_ at *A*_0_ ≤ 10 (Supplementary Fig. 7b). Most importantly, the transient emergence of the bow-tie network is completely independent of the goal rank (Supplementary Fig. 7c–e), which indicates that the determinant of the bow-tie emergence is the initial value ${A}_{0}$ and the goal variances.

### Analysis of bow-tie architecture evolution by the dynamical system

To determine the reason for the dependence on the initial link intensities ${A}_{0}$, we consider a phenomenological ordinary differential equation (ODE) of the network ${A}_{{ij}}^{\left(l\right)}$ that qualitatively mimics the evolutionary simulation described above. Based on the gradient descent, the time evolution of link intensity ${A}_{{ij}}^{\left(l\right)}$ is given by

1 $$\frac{d{A}_{{ij}}^{\left(l\right)}}{{dt}}={\eta A}_{{ij}}^{\left(l\right)}\frac{\partial F}{\partial {A}_{{ij}}^{\left(l\right)}}{\boldsymbol{,}}$$

where the product rule mutation is implemented by the learning rate proportional to ${A}_{{ij}}^{\left(l\right)}$. This optimization algorithm, which we refer to here as the multiplicative gradient descent, describes the multiplicative changes in the link intensity, and therefore the larger link changes more drastically. The learning rate $\eta$ is set to a small constant value $\eta \,={10}^{-4}$ to avoid the computational instability. We confirmed that the results obtained using the ODE model (Supplementary Fig. 8) were almost the same as those by the original model (Fig. 3)—namely, the ODE model reproduced bow-tie architectures for a rank-deficient goal matrix ***G*** and a small ${A}_{0}$. We also confirmed that the ODE model exhibited a dependence on ${A}_{0}$ similar to that of the original model (Supplementary Fig. 8a, b). The ODE model also recapitulated the transient appearance of the bow-tie in the original model (Supplementary Fig. 8c, d).

Using this ODE model, we here analyzed how the bow-tie architecture emerges in the early stage of the time evolution when starting from a small ${A}_{0}$. For simplicity, we consider networks consisting of three layers with two nodes each (Supplementary Fig. 9a) with the most straightforward rank 1 goal matrix ***G*** defined as

***G*** = $$\left[\begin{array}{cc}g & g\\ g & g\end{array}\right]$$, where $g$ takes an arbitrary positive value $\left({g}\, >\, 0\,\right).$

The following argument can also be generalized for an arbitrary ***G*** with rank 1 or rank 2 (Supplementary Note 2). Under this condition, the evaluation function is given as follows (Eq. 2):

2 $$F=-{\left\Vert{{\bf{A}}}^{\left(2\right)}{{\bf{A}}}^{\left(1\right)}-{\bf{G}}\right\Vert}_{F}^{2}=-{\rm{Tr}}\left[\left({{\bf{A}}}^{\left(2\right)}{{\bf{A}}}^{\left(1\right)}-{\bf{G}}\right){\left({{\bf{A}}}^{\left(2\right)}{{\bf{A}}}^{\left({1}\right)}-{\bf{G}}\right)}^{{\rm{T}}}\right]$$

The adaptation process is modeled using the multiplicative gradient descent in Eq. (1) as follows (Supplementary Note 1):

3 $$\left\{\begin{array}{c}\displaystyle\frac{d}{{dt}}{{\bf{A}}}^{(2)}=-2\eta \left[\left({{\bf{A}}}^{\left(2\right)}{{\bf{A}}}^{\left(1\right)}-{\bf{G}}\right){{\bf{A}}}^{\left(1\right),{\rm{T}}}\right]\odot {{\bf{A}}}^{\left(2\right)}\\ \displaystyle\frac{d}{{dt}}{{\bf{A}}}^{(1)}=-2\eta \left[{{\bf{A}}}^{\left(2\right),{\rm{T}}}\left({{\bf{A}}}^{\left(2\right)}{{\bf{A}}}^{\left(1\right)}-{\bf{G}}\right)\right]\odot {{\bf{A}}}^{\left(1\right)}\end{array}\right.$$

where $\odot$ is the Hadamard product, i.e., ${({\bf{A}}\odot {\bf{B}})}_{{ij}}={a}_{{ij}}{b}_{{ij}}$, and ${{\bf{A}}}^{\left(l\right),{\rm{T}}}$ is the transposed matrix of ${\bf A}^{\left(l\right)}$. Since we consider the early stage of the dynamics starting from ${{\bf{A}}}^{\left(1\right)}$ and ${{\bf{A}}}^{\left(2\right)}$ that are much smaller than ${\bf{G}}$, the term $\left({{\bf{A}}}^{\left(2\right)}{{\bf{A}}}^{\left(1\right)}-{\bf{G}}\right)$ can be approximated as$\,-{\bf{G}}$, and thus Eq. (3) is simplified as follows:

4 $$\frac{d}{{dt}}\left(\begin{array}{c}{{\bf{A}}}^{\left(2\right)}\\ {{\bf{A}}}^{\left(1\right),{\rm{T}}}\end{array}\right)=2\eta \left[\left(\begin{array}{cc}{\bf{0}} & {\bf{G}}\\ {{\bf{G}}}^{{\rm{T}}} & {\bf{0}}\end{array}\right)\left(\begin{array}{c}{{\bf{A}}}^{\left(2\right)}\\ {{\bf{A}}}^{\left(1\right),{\rm{T}}}\end{array}\right)\right]\odot \left(\begin{array}{c}{{\bf{A}}}^{\left(2\right)}\\ {{\bf{A}}}^{\left(1\right),{\rm{T}}}\end{array}\right)$$

By expanding Eq. (4), the following equation is obtained:

5 $$\frac{d}{{dt}}\left(\begin{array}{cc}\begin{array}{c}\begin{array}{c}{A}_{11}^{\left(2\right)}\\ {A}_{21}^{\left(2\right)}\end{array}\\ \begin{array}{c}{A}_{11}^{\left(1\right)}\\ {A}_{12}^{\left(1\right)}\end{array}\end{array} & \begin{array}{c}\begin{array}{c}{A}_{12}^{\left(2\right)}\\ {A}_{22}^{\left(2\right)}\end{array}\\ \begin{array}{c}{A}_{21}^{\left(1\right)}\\ {A}_{22}^{\left(1\right)}\end{array}\end{array}\end{array}\right)=2\eta \left(\begin{array}{cc}\begin{array}{c}\begin{array}{c}g\left({A}_{11}^{\left(1\right)}+{A}_{12}^{\left(1\right)}\right){A}_{11}^{\left(2\right)}\\ g\left({A}_{11}^{\left(1\right)}+{A}_{12}^{\left(1\right)}\right){A}_{21}^{\left(2\right)}\end{array}\\ \begin{array}{c}g\left({A}_{11}^{\left(2\right)}+{A}_{21}^{\left(2\right)}\right){A}_{11}^{\left(1\right)}\\ g\left({A}_{11}^{\left(2\right)}+{A}_{21}^{\left(2\right)}\right){A}_{12}^{\left(1\right)}\end{array}\end{array} & \begin{array}{c}\begin{array}{c}g\left({A}_{21}^{\left(1\right)}+{A}_{22}^{\left(1\right)}\right){A}_{12}^{\left(2\right)}\\ g\left({A}_{21}^{\left(1\right)}+{A}_{22}^{\left(1\right)}\right){A}_{22}^{\left(2\right)}\end{array}\\ \begin{array}{c}g\left({A}_{12}^{\left(2\right)}+{A}_{22}^{\left(2\right)}\right){A}_{21}^{\left(1\right)}\\ g\left({A}_{12}^{\left(2\right)}+{A}_{22}^{\left(2\right)}\right){A}_{22}^{\left(1\right)}\end{array}\end{array}\end{array}\right)$$

Note that the first and second columns are independent because the time evolution of ${({A}_{11}^{\left(2\right)},{A}_{21}^{\left(2\right)},{A}_{11}^{\left(1\right)},{A}_{12}^{\left(1\right)})}^{{\rm{T}}}$ does not depend on the ${({A}_{12}^{\left(2\right)},{A}_{22}^{\left(2\right)},{A}_{21}^{\left(1\right)},{A}_{22}^{\left(1\right)})}^{{\rm{T}}}.$ The first column of Eq. (5) is given as

6 $$\frac{d}{{dt}}\left(\begin{array}{c}\begin{array}{c}{A}_{11}^{\left(2\right)}\\ {A}_{21}^{\left(2\right)}\end{array}\\ \begin{array}{c}{A}_{11}^{\left(1\right)}\\ {A}_{12}^{\left(1\right)}\end{array}\end{array}\right)=2\eta \left(\begin{array}{c}\begin{array}{c}g({A}_{11}^{\left(1\right)}+{A}_{12}^{\left(1\right)}){A}_{11}^{\left(2\right)}\\ g({A}_{11}^{\left(1\right)}+{A}_{12}^{\left(1\right)}){A}_{21}^{\left(2\right)}\end{array}\\ \begin{array}{c}g({A}_{11}^{\left(2\right)}+{A}_{21}^{\left(2\right)}){A}_{11}^{\left(1\right)}\\ g({A}_{11}^{\left(2\right)}+{A}_{21}^{\left(2\right)}){A}_{12}^{\left(1\right)}\end{array}\end{array}\right)$$

Here, we define ${I}_{k}$ and ${R}_{k}$ as follows.

7 $$\begin{array}{c}{I}_{k}={A}_{k1}^{\left(1\right)}+{A}_{k2}^{\left(1\right)}\\ {R}_{k}={A}_{1k}^{\left(2\right)}+{A}_{2k}^{\left(2\right)}\end{array}$$

${I}_{k}$ represents the sum of the input link intensities to the node *k* in the middle layer, whereas ${R}_{k}$ denotes the sum of the output intensities from node *k* in the middle layer (Supplementary Fig. 9a). From Eqs. (6) and (7), the following relation holds:

8 $$\begin{array}{c}\displaystyle\frac{d}{{dt}}{I}_{k}=2\eta g{R}_{k}{I}_{k}\\ \displaystyle\frac{d}{{dt}}{R}_{k}=2\eta g{R}_{k}{I}_{k}\end{array}$$

Thus, $\frac{d}{{dt}}\left({R}_{k}-{I}_{k}\right)=0,$ indicating ${R}_{k}-{I}_{k}={R}_{k,0}-{I}_{k,0}$, where ${R}_{k.0}$ and ${I}_{k,0}$ are the initial values of ${R}_{k}$ and ${I}_{k}$ (Supplementary Fig. 9b). Substituting this expression into Eq. (8), $\frac{d}{{dt}}{R}_{k}$ is described as follows.

9 $$\frac{d}{{dt}}{R}_{k}=2\eta g{R}_{k}\left({R}_{k}-{R}_{k,0}+{I}_{k,0}\right)$$

By solving this, the time evolution of ${R}_{k}$ is derived.

10 $${R}_{k}\left(t\right)=\frac{{R}_{k,0}\left({R}_{k,0}-{I}_{k,0}\right)}{{R}_{k,0}-{I}_{k,0}\exp \left[2\eta g\left({R}_{k,0}-{I}_{k,0}\right)t\right]}$$

This indicates that ${R}_{k}$ diverges within a finite time, ${t}=\frac{1}{2\eta g}\frac{\,\mathrm{ln}{R}_{k,0}-\mathrm{ln}{I}_{k,0}}{{R}_{k,0}-{I}_{k,0}}$, and ${I}_{k}$ also diverges since ${R}_{k}-{I}_{k}={const}.$ The same argument holds for the second column of Eq. (5), since the equations for both columns are exactly symmetric and independent of each other. However, due to the difference in the initial value, one of the two columns diverges prior to the other, and at this moment the network forms a bow-tie structure. Once this divergence of one of the two columns occurs, the time evolution significantly slows down and the diverging nature in the dynamics vanishes since the assumption ${\bf{G}}-{\bf{A}}\,\approx\, {\bf{G}}$ does not hold, which indicates that the transiently emerging bow-tie architecture is maintained. An example of the dynamics is shown in supplementary Fig. 9b, where ${R}_{1}$ and ${I}_{1}$ diverge (Supplementary Fig. 9b; right panels) and accordingly, the bow-tie architecture associated with node 1 is formed (Supplementary Fig. 9b; left panels). For the case of ${R}_{k,0}\,\approx\, {I}_{k,0}$, the divergent time is written as $t \sim 1/g{R}_{k,0}$ (Supplementary Note 3), which indicates that the column that has a larger ${R}_{k,0}$ diverges first.

Although the rank 1 goal matrix with 2 nodes is assumed here, the independent relationships between columns of $$\left(\begin{array}{c}{{\bf{A}}}^{\left(2\right)}\\ {{\bf{A}}}^{\left(1\right),{\rm{T}}}\end{array}\right)$$ in Eq. (4) hold, regardless of the goal matrix rank and number of nodes (Supplementary Note 2). Therefore, the bow-tie emergence with the divergence of $R$ and $I$ for one of the columns always occurs if the approximation ${\bf{G}}-{\bf{A}}\,\approx\, {\bf{G}}$ is valid. The intuitive explanation for this effect is as follows: In the case of ${\bf{G}}-{\bf{A}}\,\approx\, {\bf{G}}$, all ${A}_{{ij}}^{\left(l\right)}$ grow to increase the fitness, but the multiplicative nature of the growth leads to the significant difference ${A}_{{ij}}^{\left(l\right)}$ among the *l*th layer; i.e., ${A}_{{ij}}^{\left(l\right)}$ starting from the smaller value grows more slowly, which causes the bow-tie architecture. This is not the case for the condition under which the system starts from a large initial value $\|{\bf{A}}\|_{F}\,\approx\, \|{\bf{G}}\|_{F}$, because the fitness reaches the optimal value before the product rule mutation causes a significant difference of ${A}_{{ij}}^{\left(l\right)}$. These arguments help to explain how the bow-tie architecture emerges in the early phase of evolution when the initial link intensities are small, regardless of the goal rank.

### Environmental fluctuation facilitates the emergence of the bow-tie architecture

**Fig. 4:**
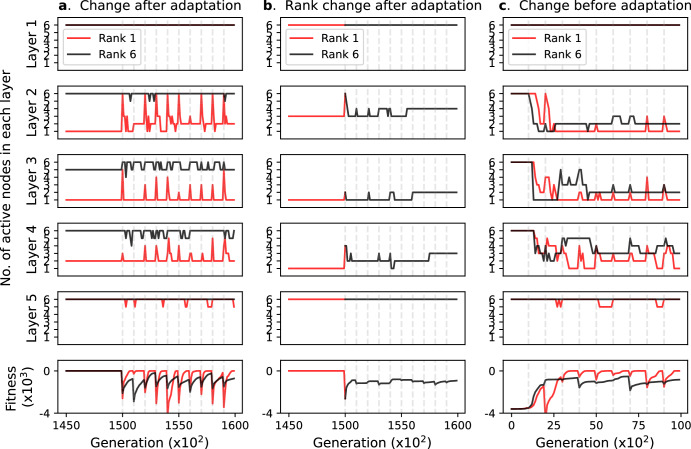
Introduction of goal matrix fluctuation in a network with M = 6 L = 4 (5 layers). The goal matrix is changed every 1000 generations (dashed line) after or before the adaptation. The number of nodes in each layer (upper 5 panels) and the fitness of the most-adapted network in the population (the bottom panel) are plotted against the generation. **a** Sequential goal matrix changes after fitness is converged. The goal matrix changes without changing the norm and rank (red: rank 1; black: rank 6). **b** Sequential goal matrix changes after fitness is converged. The goal matrix rank is changed from rank 1 to rank 6 in the first change. **c** Sequential goal matrix changes from the beginning of evolution. The goal matrix rank and norm do not change (red: rank 1; black: rank 6).

The aforementioned results assume a fixed goal matrix, which corresponds to the evolution under a fixed environment. However, in a more realistic situation, the elements of the goal matrix (ideal in-out relation) fluctuate due to the environmental fluctuation or mutations in the numbers of the receptors and downstream genes. To examine the effect of an environmental fluctuation on the evolution of bow-tie architecture, we introduced the variable goal matrix without changing the dimension, rank, or norm. First, the network evolves under the first goal matrix for 150,000 generations, which is enough time for the fitness to reach 0.01. Second, the goal matrix is altered to the next one. We found that the network architectures are maintained irrespective of any such change in the goal matrix during evolution; the waist width did not change between before and after the change in goal matrix (Supplementary Fig. 10), indicating that the waist width is not affected by the change in goal matrix but determined by ${A}_{0}$. The number of active nodes transiently increases immediately after the change of goal matrix. This is attributed to the definition of active nodes; due to the decrease in fitness associated with the change in the goal matrix, the difference of $\Delta {F}_{i}^{l}$ (i.e., the fitness decrease by the *l*th node removal) among nodes in the *l*th layer is less clear, and thus the value of ${P}_{i}^{l}=\Delta {F}_{i}^{l}/{\sum }_{i}^{M}\Delta {F}_{i}^{l}$ tends to exceed the threshold, which leads to an increase in the waist width. These results reflect that the bow-tie architectures are robust to environmental fluctuations that do not alter the rank and norm once the bow-tie architecture emerges. Consistent with such a conclusion, after the network adapts to the initial goal, the network architecture does not change by repeated changes in the goal matrix. Figure 4a shows the time-course of the active nodes in each layer during the repeated goal change with a fixed goal rank (ranks 1 and 6) every 1000 generations, indicating that the network architecture is maintained (for ranks 2 and 3, see Supplementary Fig. 11a). Interestingly, after the adaptation to the goal matrix, the network structure is still maintained even for the changes in the rank of the goal matrix. The network is first evolved to adapt to the rank 1 goal, and then the goal matrix is repeatedly switched to the randomly generated full-rank goal matrix every ${10}^{3}$ generation (Fig. 4b). This procedure did not change the bow-tie architecture during ${10}^{4}$ generations. Similarly, the retention of bow-tie architecture was also observed in the changes from rank 2 to rank 6 and from rank 3 to rank 6 (Supplementary Fig. 11b).

Although these data indicate that the environmental fluctuation does not affect the previously emerged bow-tie architecture, this is not the case when the fluctuation takes place at the beginning of the evolution. When starting from a small initial link intensity ${A}_{0}$ (${A}_{0}=0.01$), the initial random network develops the bow-tie architecture by the repeated fluctuation of the goal matrix, regardless of the goal rank (Fig. 4c and Supplementary Fig. 11c). This can be understood by the fact that the time-averaged goal matrix can be effectively interpreted as a rank 1 matrix, which facilitates the bow-tie emergence.

### Gene duplication accelerates the emergence of the bow-tie architecture

**Fig. 5:**
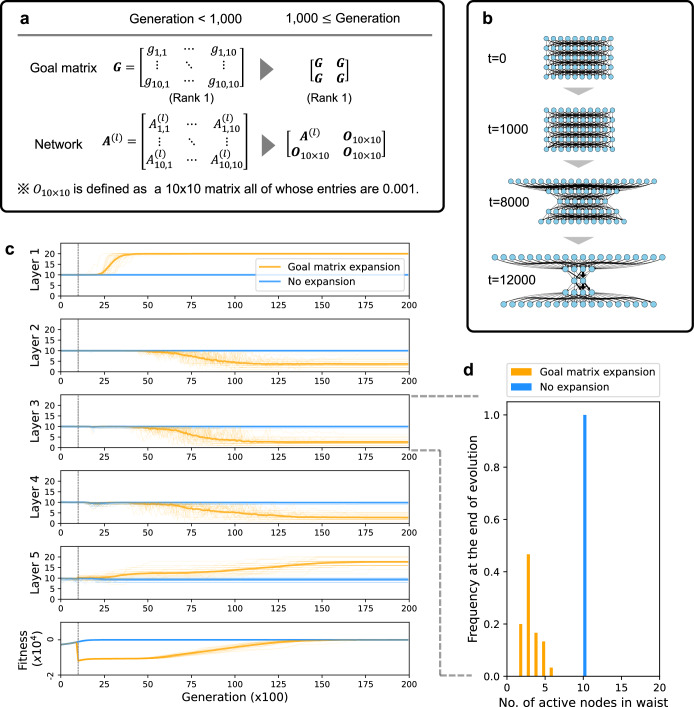
Expansion of the goal matrix drives evolution of the bow-tie architecture. The procedure of goal matrix expansion. **a** The rank 1 10 × 10 goal matrix G is expanded to the 20 × 20 matrix at the 1000th generation, maintaining the matrix rank 1. In association with the goal matrix expansion, the network matrix ${{\bf{A}}}^{{\boldsymbol{(}}{{l}}{\boldsymbol{)}}}$ is also expanded to a 20 × 20 matrix by padding using the near zero 10 × 10 matrix ${{\bf{O}}}_{10\times 10}$. **b** An example of the network during evolution after the goal expansion. **c** Evolutionary trajectories of the network. X-axis: Generation. Y-axis: Number of nodes in each layer. The initial norm ${A}_{0}$ is 10. The orange line represents the case where the 10 × 10 goal matrix with rank 1 is expanded to 10 × 10 at the 1000th generation (dashed line), while the blue line represents the simulation with no enlargement. Trajectories are averaged among independent simulation runs (*n* = 100 for each color). **d** Statistics of the number of active nodes in the adapted network for each layer (x-axis) with (orange) or without (blue) the goal matrix expansion (*n* = 100 for each color).

We next examined the effect of the expansion of the goal matrix. During evolution, the number of the input and output nodes can be increased by gene duplication, and by de-novo addition (emergence) of the receptor molecules and downstream genes. Therefore, we investigated whether the non-bow-tie network switches to the bow-tie architecture in response to an increase in the goal matrix dimension. We found that an increase in the goal matrix dimension indeed leads to the emergence of the bow-tie architecture even when starting from a large link intensity (Fig. 5). The details of the simulation procedure are shown in Fig. 5a: Evolutionary simulations were started from a network including 10 nodes by 5 layers with a large initial link intensity (${{\rm{A}}}_{0}=10)$ under a rank 1 goal. Since the simulation started from a large ${{\rm{A}}}_{0}$, the network did not evolve to a bow-tie network. Nevertheless, by expanding the goal matrix from a 10 × 10 matrix to a 20 × 20 matrix at the 1000th generation (Fig. 5a), the waist width was reduced, resulting in the convergence (|Fitness| < 0.01) to the bow-tie architecture with a narrower waist (Fig. 5b,c). Note that although the input and output nodes are increased by the goal matrix expansion, the evolved waist width is lower than that before expanding the goal matrix (Fig. 5c, d). This would also be explained from the proposed scenario starting from a small link intensity; the expansion of the goal matrix results in the increase in the norm of the goal matrix ${\bf{G}},$ causing$\,\|{\bf{G}}\|_{F}\gg \|{\bf{A}}\|_{F}$, and subsequently leads to evolution of the bow-tie architecture as discussed in the above section.

### Bow-tie network with non-linear interactions

Although we considered a network with linear interactions in this study, actual biological interactions are often non-linear. Hence, we examined whether the bow-tie architecture emerges in the ${A}_{0}$ dependent manner even when the interactions are non-linear. In a non-linear network, the activation function $f\left(x\right)=(1+\tanh ({\rm{x}}))/2$ is introduced into each layer. The network evolves to solve a classification task of the 64-bit image of a handwritten digit. The input ${\bf{v}}$ is a 64-length array that represents the 2D (8×8) image of a handwritten number (the digit dataset in scikit-learn). The handwritten numbers in the input images are “2”, “4”, “6” and “8”, and these images are classified into the redundant six groups (see Supplementary Fig. 13a), which is encoded to a 6-bit array of the output.

The network consists of 5 layers (64 nodes for the input layer and 6 nodes for the other layers). The network output is described as ${\bf{u}}={f}({\bf{A}}^{\left(4\right)}f({\bf{A}}^{\left(3\right)}{{f}({\bf{A}}}^{\left(2\right)}f({{\bf{A}}}^{\left(1\right)}{\bf{v}})))$, where ${\bf{u}}$ and ${\bf{v}}$ are the output and input vectors, while ${\bf{A}}^{(1)}\in {R}_{6\times 64}$ and ${\bf{A}}^{\left(i\right)}\in {R}_{6\times 6},{i}={2,3,4}$ (Supplementary Fig. 13a). By genetic algorithm with the multiplicative mutations, the network was trained to solve the classification problem for the image inputs (300 images). In the initial condition, ${A}_{{ij}}^{\left(l\right)}$ is extracted from the uniform distribution from −1 to 1, followed by the normalization of the norm of ${\bf{A}}={\bf{A}}^{\left(4\right)}{\bf{A}}^{\left(3\right)}{\bf{A}}^{\left(2\right)}{\bf{A}}^{\left(1\right)}$ to ${A}_{0}$. The mutation size is set to large so that the intensities can take both negative and positive values (i.e., $\xi \sim N(1,\,0.5)$). Supplementary Fig. 13b shows the time trajectory of the number of the active nodes in each layer. The networks starting from small link intensities ${A}_{0}$ (Supplementary Fig. 13b: solid line) tended to have a narrower middle layer in both the transient and end state than those starting from a large ${A}_{0}$ (Supplementary Fig. 13b: dashed line). This suggests that the initial value plays a crucial role in the evolution of bow-tie architecture, even in a non-linear network.

## Discussion

In this study, we investigated the conditions under which the bow-tie architecture emerges by using an evolutionary simulation with a linear network model. We found that the prime determinant of the bow-tie emergence is the link intensity of the initial network ${A}_{0}$ rather than characteristics in the goal matrix. In the end state of evolution, only when evolution starts from small ${A}_{0}$, the network can evolve into a bow-tie architecture in a goal matrix rank-dependent manner. In the transient state of evolution, when evolution starts from small ${A}_{0}$, bow-tie architecture always emerges independently from the goal matrix rank. Namely, the bow-tie emerges spontaneously when ${A}_{0}$ is small, and the rank of the goal matrix determines whether or not the transiently emerged bow-tie will be maintained until the end state. From the analysis using the ODE model (Fig. 4), we concluded that this emergence of bow-tie architecture is attributable to the growth from a small value of ${A}_{0}$ with the multiplicative nature of the increase in ${A}_{{ij}}^{\left(l\right)}$, which can cause the significant differences in ${A}_{{ij}}^{\left(l\right)}$ among the *l*th layers. Our result indicates that the initial value plays a crucial role in the evolution of bow-tie architecture rather than the goal matrix, which was overlooked in the previous study^2^.

The evolution from small ${A}_{0}$ (i.e., small link intensity) may correspond to the early stage in the evolution, where molecular interactions are almost entirely absent. Consistent with such an assumption that the bow-tie network could emerge at the early stage of the evolution, a core of the bow-tie architecture is well conserved (e.g., G proteins), implying that the architecture has a very old origin^19,36,37^. On the other hand, the evolution from a large ${A}_{0}$ corresponds to a readapting after the network evolves to a non-bow-tie architecture. In this case, under a new goal, the network does not attain a bow-tie structure unless the condition $\|{\bf{G}}\|_{F}\gg {A}_{0}$ is satisfied for the new goal. For the case with $\|{\bf{G}}\|_{F}\gg {A}_{0}$, the bow-tie architecture emerges after the goal matrix changes to the large norm goal matrix (Fig. 5).

It is known that the bow-tie architecture is robust against perturbation^1,2,23^ and thus the core is well conserved^19,36,37^. Consistent with these facts, we confirmed that once the bow-tie architecture is established, it is robust against the goal matrix fluctuations (Fig. 4a, b). Additionally, we found that continuous change of the goal matrix stabilizes the bow-tie architecture (Fig. 4c).

The vast number of inputs and outputs is also characteristic of bow-tie architecture. Thus we examined the relationship between the expansion event of input/output (i.e., genome duplication, de novo addition) and bow-tie evolution. We found that a sudden increase in the number of inputs and outputs narrowed the waist width in the network, leading to the bow-tie architecture, even though the adaptation process started from a relatively large initial condition. In addition, the previous study^2^ showed that once a bow-tie architecture is established, it is robust to further goal matrix expansion (Supplementary Fig. 17 in ref. ^2^). Similarly, information processing has been reported to be robust against the increase in input/output nodes^38^. These findings suggest that the addition of inputs and outputs (i.e., goal matrix expansion) does not disturb the bow-tie structure, and can even promote its emergence (Fig. 5). In fact, in the GPCR signaling system, which forms a narrow waist bow-tie architecture, the numbers of receptors and downstream genes have been reported to increase rapidly^36,39^. However, further analysis will be needed to determine whether such expansion gave rise to the formation of bow-tie architecture in actual biological systems. It is also important to address how the size of the intermediate layer grows as the network size increases and to examine the presence or absence of the scaling law in these growth curves, as was observed in the archaea genome^40^.

Our results indicate that the bow-tie architecture can emerge in a goal-independent manner. This suggests a possibility that the bow-tie architecture emerges as a byproduct of evolution, where a phenotype can emerge regardless of the evolutionary goal. However, it does not deny the possibility that the bow-tie architecture emerges as the result of selection for the functional advantages suggested by the previous studies^17,26–28^ or as the result of the rank-deficient task^2^. For example, the bow-tie architecture can be controlled by controlling input signals^27^, and it shows fast adaptation to novel environments and high evolvability ^28^. Such fast adaptation of the bow-tie architecture was also seen in our simulation (Fig. 4a, red line in the bottom panel). We also found that the network generally evolves to a bow-tie architecture under the fluctuating goal matrix (Fig. 4c and Supplementary Fig. 11c). This suggests that the bow-tie structure can provide a fast adaptation to a fluctuating environment. On the other hand, the bow-tie network has a high lethality to perturbation to the core nodes^41,42^ and is disadvantageous for use in a search for alternative core genes^23^. The bow-tie architecture would have been selected as the result of a trade-off between these advantages and disadvantages^23^. The cost of having interactions could also promote the bow-tie structure. For example, when the cost of the L1 regularization term $\lambda \mathop{\sum }\limits_{l=1}^{L}\mathop{\sum }\limits_{i,j=1}^{M}|{A}_{{ij}}^{(l)}|$ is included in the fitness, the networks evolve towards the bow-tie architecture (Supplementary Fig. 12) independent of the initial link intensities *A*_*0*_, while the cost of the L2 regularization term $\lambda \mathop{\sum }\limits_{l=1}^{L}\mathop{\sum }\limits_{i,j=1}^{M}{\left({A}_{{ij}}^{(l)}\right)}^{2}$ cannot enhance the bow-tie structure (Supplementary Fig. 12). These results are explainable by the well-known effect in the data sciences^43^ that the L1 regularization term reduces the network links. However, it is not obvious whether the cost for ${A}_{{ij}}$ can be approximated by L1, L2 or other types of regularization. Thus, it would be important to consider a cost-independent scenario, as was demonstrated in this study.

Bow-tie architecture is also closely related to the neural network. The linear network model that we adopted corresponds to a neural network with a linear activation function. Even in this linear model, behaviors analogous to widely used neural networks have been reported^44,45^. The evolutionary simulation of a non-linear network performed herein (Supplementary Fig. 13) corresponds to an image classification by a genetic algorithm-optimized neural network. Further, neural networks incorporating bottlenecks in the hidden layers, such as VAE (Variational Autoencoder), are employed for tasks such as dimension reduction. Thus, a comparison of neural networks and the biological bow-tie architecture might yield interesting insights. For example, from this aspect, the information-theoretic principle^38^ and classification ability^17^ have been evaluated in signaling pathways.

In summary, we found that a key determinant of the bow-tie emergence is the intensity of molecular interactions, ${A}_{0}$, at the initial stage of the evolution, and the bow-tie structure appears if the mathematical condition$\,\|{\bf {G}}\|_{{\rm{F}}}\gg {A}_{0}$ (i.e., a large gap between the initial and goal link intensities) is satisfied. This condition would hold in (1) the early stage of evolution and (2) evolution after an addition of input and output nodes. Furthermore, the transiently appearing bow-tie structure is maintained under a fluctuating evolutionary goal. Although a feedforward network was adopted here, a more realistic interaction including feedback loops should be examined in the future. Finally, a bioinformatic analysis revealing the origin and evolutionary history of bow-tie architecture will be required to support our hypothesis.

## Methods

### The definitions of the active node

In our simulation, we mainly used the definition of the active node based on the relative fitness contribution (the 1st definition below), while we also confirmed that the obtained results do not change by using other definitions of the active node, such as that based on the relative contribution to the total in-out relation (the 2nd definition below), and that based on the relative strength of maximal interaction (the 3rd definition below). The details of the definitions of the active nodes are as follows:

#### Relative contribution of each node to the fitness

The active nodes are determined by evaluating the following ${P}_{i}^{l}$ for all nodes:

11 $${P}_{i}^{l}=\frac{{{\rm{||}}\Delta {F}_{i}^{l}{\rm{||}}}_{F}^{2}}{{\sum }_{j}^{M}{{||}\Delta {F}_{j}^{l}{||}}_{F}^{2}}$$

where $\Delta {F}_{i}^{l}$ is a fitness decrease by the deletion of the node $i$ in layer $l$. The node that shows ${P}_{i}^{l}\, >\, 0.001$ is categorized as the active node.

#### Relative contribution of each node to the total in-out relation

The active nodes are determined by evaluating the following ${P}_{i}^{l}$ for all nodes:

12 $${P}_{i}^{l}=\frac{{{\rm{||}}{{\Delta }}{{\bf{A}}}_{i}^{l}{\rm{||}}}_{F}^{2}}{{\sum }_{j}^{M}{{\rm{||}}\Delta {{\bf{A}}}_{j}^{l}{\rm{||}}}_{F}^{2}}$$

where $\Delta {{\bf{A}}}_{i}^{l}={\bf{A}}-\,{{\bf{A}}}_{{\rm{del}},{i}}^{l}\,$. ${{\bf{A}}}_{{\rm{del}},{i}}^{l}$ is a total in-out relation when node $i$ in layer $l$ is deleted. The node that shows ${P}_{i}^{l}\, >\, 0.001$ is categorized as the active node.

#### Relative strength of maximal interaction of each node

The active nodes are determined by evaluating the following ${P}_{i}^{l}$ for all nodes:

13 $${P}_{i}^{l}=\frac{{m}_{i}^{l}}{{\sum }_{j}^{M}{m}_{j}^{l}}$$

where ${m}_{i}^{l}$ describes the maximum interactions associated with node $i$ among inputs ${A}_{{ik}}^{l-1}$ and outputs ${A}_{{ki}}^{l}$, i.e., ${m}_{i}^{l}=\max ({A}_{i1}^{l-1},\ldots ,{A}_{{iM}}^{l-1},\,{A}_{1i}^{l},\ldots ,{A}_{{Mi}}^{l})$. The node that shows ${P}_{i}^{l}\, >\, 0.05$ is categorized into the active node.

### Goal matrix normalization of variance and norm

The normalization of the variance and norm of each of the elements in the $N\times N$ goal matrix is performed using the following equation:

14 $${Z}_{{ij}}=\,\frac{{G}_{{ij}}-\,{\boldsymbol{ < }}\,{G}_{{ij}}{\boldsymbol{ > }}}{\sqrt{{V}_{{G}_{{ij}}}}}+\,\sqrt{\frac{D}{{N}^{2}}-\,1},$$

where ${\boldsymbol{ < }}\,{G}_{{ij}}{\boldsymbol{ > }}$ is the average of elements in matrix ***G*** defined by ${\boldsymbol{ < }}{G}_{{ij}}{\boldsymbol{ > }}\,{\boldsymbol{=}}\,\frac{1}{{N}^{2}}{\sum }_{i}^{N}{\sum }_{j}^{N}{G}_{{ij}}$, while ${V}_{{G}_{{ij}}}$ is the variance of elements defined by ${V}_{{G}_{{ij}}}=\frac{1}{{N}^{2}}{\sum }_{i}^{N}{\sum }_{j}^{N}{\left({G}_{{ij}}- < {G}_{{ij}} > \right)}^{2}$. Goal matrix ***G*** is a random matrix with arbitrary rank. The normalized goal matrix ***Z*** shows variance ${V}_{{Z}_{{ij}}}=1$ and norm $\|{\bf{Z}}\|_{F}^{2}=D$ as shown in the following derivation.

Variance:

15 $${V}_{{Z}_{{ij}}}=\frac{1}{{N}^{2}}\mathop{\sum }\limits_{i}^{N}\mathop{\sum }\limits_{j}^{N}{\left({Z}_{{ij}}- <\, {Z}_{{ij}}\, > \right)}^{2}=\frac{1}{{{V}_{{G}_{{ij}}}N}^{2}}\mathop{\sum }\limits_{i}^{N}\mathop{\sum }\limits_{j}^{N}{\left({G}_{{ij}}-\, < {G}_{{ij}}\, > \right)}^{2}=1.$$

Norm:

16 $$\begin{array}{c}{{{||}}{\bf{Z}}{{||}}}_{F}^{2}=\mathop{\sum }\limits_{i}^{N}\mathop{\sum }\limits_{j}^{N}{{Z}_{{ij}}}^{2}={N}^{2}(V_{{Z}_{{ij}}}+\,{ <\, {Z}_{{ij}}\, > }^{2})\\\quad\; ={N}^{2}\left(1+\,{\left(\sqrt{\displaystyle\frac{D}{{N}^{2}}-1}.\right)}^{2}\right)=D,\end{array}$$

where $<\, {{Z}_{{ij}}}^{2}\, > =\,{V}_{{Z}_{{ij}}}+\,{ <\, {Z}_{{ij}}\, > }^{2}$ is used.

### Supplementary information

Supplementary Material

## Data Availability

All data used in this study can be freely downloaded from Zenodo (10.5281/zenodo.11090260).

## Code availability

Code files are freely available from the GitHub repository (https://github.com/itotoma/BowTie_evolution.git).
