# Supplementary material for: Revisiting the evolution of bow-tie architecture in signaling networks
Source: NPJ Syst Biol Appl. 2024 Jun 29;10:70. doi: 10.1038/s41540-024-00396-8 (PMC11217396; doi:10.1038/s41540-024-00396-8)
Supplement: Supplementary file 1 — Supplementary Material [file 41540_2024_396_MOESM1_ESM.pdf]

Supplementary Materials for
"Revisiting the Evolution of Bow-tie Architecture in Signaling Networks"

Thoma Itoh[1,2,3], Yohei Kondo[1,2,3], Kazuhiro Aoki[1,2,3], Nen Saito*[3,4]

[1] National Institute for Basic Biology, National Institutes of Natural Sciences, 5-1 Higashiyama, Myodaiji-cho, Okazaki, Aichi, 444-8787, Japan
[2] Department of Basic Biology, School of Life Science, SOKENDAI (The Graduate University for Advanced Studies), 5-1 Higashiyama, Myodaiji-cho, Okazaki, Aichi, 444-8787, Japan
[3] Exploratory Research Center on Life and Living Systems (ExCELLS), National Institutes of Natural Sciences, 5-1 Higashiyama, Myodaiji-cho, Okazaki, Aichi, 444-8787, Japan
[4] Graduate School of Integrated Sciences for Life, Hiroshima University, Higashihiroshima, Hiroshima 739-8511, Japan

Supplementary Note 1. **The ODE model for bow-tie architecture evolution.**

To analyze evolutionary dynamics of the linear network model, we here consider a phenomenological ordinary differential equation (ODE) of the network that qualitatively mimics the evolutionary simulation described in the main text. The linear network model that is defined in the main text consists of L-1 $M \times M$ matrices $\{\boldsymbol{A}^{(\boldsymbol{1})}\ldots\boldsymbol{A}^{(\boldsymbol{L-1})}\}$ and describes a layered feedforward network with M nodes in each layer. The link intensities from node j in the l th layer to node i in the $l+1$ th layer are described by $\boldsymbol{A}^{(\boldsymbol{l})}$ as

$$\boldsymbol{A}^{(\boldsymbol{l})} = \begin{pmatrix} A_{11}^{(l)} & \cdots & A_{1M}^{(l)} \\ \vdots & \ddots & \vdots \\ A_{M1}^{(l)} & \cdots & A_{MM}^{(l)} \end{pmatrix}$$

The time evolution of the link intensity $A_{ij}^{(l)}$ is given by eq.1 in the main text as a proxy of the evolutionary simulation and can be described in the follow matrix form:

$$\frac{d\boldsymbol{A}^{(l)}}{dt} = \eta \frac{\partial F}{\partial A^{(l)}} \odot \boldsymbol{A}^{(l)} \tag{1}$$

The evaluation function F is given as

$$F = -\|\boldsymbol{A} - \boldsymbol{G}\|_2 = -\mathrm{Tr}[(\boldsymbol{A} - \boldsymbol{G})(\boldsymbol{A} - \boldsymbol{G})^{\mathrm{T}}]. \tag{2}$$

Here, A is the total in-out relation matrix and given as

$$\boldsymbol{A} = \prod_{i=0}^{L-1} \boldsymbol{A}^{(L-i)} = \boldsymbol{A}^{(L)}\boldsymbol{A}^{(L-1)} \ldots \boldsymbol{A}^{(1)}. \tag{3}$$

The derivative of F by $A_{ij}^{(l)}$, $\frac{\partial F}{\partial A_{ij}^{(l)}}$, is given as follows:

$$\frac{\partial F}{\partial A_{ij}^{(l)}} = -2\ \mathrm{Tr}[(\mathbf{A}-\mathbf{G})\ (\boldsymbol{A}^{\mathrm{T}})']$$

$$= -2\ \mathrm{Tr}\left[(\boldsymbol{A}-\boldsymbol{G})\boldsymbol{A}^{(1),\mathrm{T}}\boldsymbol{A}^{(2),\mathrm{T}} \dots \boldsymbol{A}^{(l-1),\mathrm{T}}(\boldsymbol{A}^{(l),\mathrm{T}})'\boldsymbol{A}^{(l+1),\mathrm{T}} \dots \boldsymbol{A}^{(L),\mathrm{T}}\right]$$

$$= -2\ \mathrm{Tr}\left[\boldsymbol{A}^{(l+1),\mathrm{T}} \dots \boldsymbol{A}^{(L),\mathrm{T}}(\boldsymbol{A}-\boldsymbol{G})\boldsymbol{A}^{(1),\mathrm{T}}\boldsymbol{A}^{(2),\mathrm{T}} \dots \boldsymbol{A}^{(l-1),\mathrm{T}}(\boldsymbol{A}^{(l),\mathrm{T}})'\right]. \quad (4)$$

Here we denote $\frac{\partial X}{\partial A_{ij}^{(l)}}$ as $(X)'$. We use $(\boldsymbol{AB})^{\mathrm{T}} = \boldsymbol{B}^{\mathrm{T}}\boldsymbol{A}^{\mathrm{T}}$ and $\mathrm{Tr}[\boldsymbol{AB}] = \mathrm{Tr}[\boldsymbol{BA}]$ in the above equation.
Since $(\boldsymbol{A}^{(l)})'$ is the matrix in which only the ij element is 1 and the other elements are 0, $\mathrm{Tr}\left[\boldsymbol{X}^{T}(\boldsymbol{A}^{(l),\mathrm{T}})'\right] = X_{ij}$ holds. Then,

$$\frac{\partial F}{\partial A_{ij}^{(l)}} = -2\left[\boldsymbol{A}^{(l+1),\mathrm{T}} \dots \boldsymbol{A}^{(L),\mathrm{T}}(\boldsymbol{A}-\boldsymbol{G})\boldsymbol{A}^{(1),\mathrm{T}}\boldsymbol{A}^{(2),\mathrm{T}} \dots \boldsymbol{A}^{(l-1),\mathrm{T}}\right]_{ij}.$$

This can be described in the matrix form as follows:

$$\frac{\partial F}{\partial \boldsymbol{A}^{(l)}} = -2\ (\boldsymbol{A}^{(l+1),\mathrm{T}} \dots \boldsymbol{A}^{(L),\mathrm{T}})(\boldsymbol{A}-\boldsymbol{G})(\boldsymbol{A}^{(1),\mathrm{T}}\boldsymbol{A}^{(2),\mathrm{T}} \dots \boldsymbol{A}^{(l-1),\mathrm{T}}). \quad (5)$$

From *eq.* (5) and *eq.* (1), the time derivative of $A^{(l)}$ is

$$\frac{d\boldsymbol{A}^{(l)}}{dt} = -2\eta(\boldsymbol{A}^{(l+1),\mathrm{T}} \dots \boldsymbol{A}^{(L),\mathrm{T}})(\boldsymbol{A}-\boldsymbol{G})(\boldsymbol{A}^{(1),\mathrm{T}}\boldsymbol{A}^{(2),\mathrm{T}} \dots \boldsymbol{A}^{(l-1),\mathrm{T}}) \odot \boldsymbol{A}^{(l)}. \quad (6)$$

Equation 3 in the main text is derived by assuming $L = 2\ (l \leq 2)$ to *eq.* (6).

Supplementary Note 2. **Bow-tie evolution when the goal matrix is full rank.**

Here, we consider the 2×2 goal matrix,

$\boldsymbol{G} = \begin{bmatrix} g_1 & g_2 \\ g_3 & g_4 \end{bmatrix}$ where $0 < g_1 \leq g_2, g_3, g_4$.

For considering the early stage of evolution starting from a small initial link intensity, we assume that $\boldsymbol{A}$ is much smaller than $\boldsymbol{G}$, and thus $\boldsymbol{A}-\boldsymbol{G} \approx -\boldsymbol{G}$. The time evolution of link intensities is given as follows:

$$\frac{d}{dt}\begin{pmatrix} \boldsymbol{A}^{(2)} \\ \boldsymbol{A}^{(1),\mathrm{T}} \end{pmatrix} = 2\eta\left[\begin{pmatrix} \mathbf{0} & \boldsymbol{G} \\ \boldsymbol{G}^{T} & \mathbf{0} \end{pmatrix}\begin{pmatrix} \boldsymbol{A}^{(2)} \\ \boldsymbol{A}^{(1),\mathrm{T}} \end{pmatrix}\right] \odot \begin{pmatrix} \boldsymbol{A}^{(2)} \\ \boldsymbol{A}^{(1),\mathrm{T}} \end{pmatrix}. \quad (7)$$

The first column of the above equation can be rewritten as

$$\frac{d}{dt}\begin{pmatrix} A_{11}^{(2)} \\ A_{21}^{(2)} \\ A_{11}^{(1)} \\ A_{12}^{(1)} \end{pmatrix} = 2\eta \begin{pmatrix} \left(A_{11}^{(1)} g_1 + A_{12}^{(1)} g_3\right) A_{11}^{(2)} \\ \left(A_{11}^{(1)} g_2 + A_{12}^{(1)} g_4\right) A_{21}^{(2)} \\ \left(A_{11}^{(2)} g_1 + A_{21}^{(2)} g_2\right) A_{11}^{(1)} \\ \left(A_{11}^{(2)} g_3 + A_{21}^{(2)} g_4\right) A_{12}^{(1)} \end{pmatrix} \tag{8}$$

Since $g_1 \le g_2, g_3, g_4$, the following inequality is obtained:

$$\frac{d}{dt}\begin{pmatrix} A_{11}^{(2)} \\ A_{21}^{(2)} \\ A_{11}^{(1)} \\ A_{12}^{(1)} \end{pmatrix} \ge 2\eta \begin{pmatrix} g_1\left(A_{11}^{(1)} + A_{12}^{(1)}\right) A_{11}^{(2)} \\ g_1\left(A_{11}^{(1)} + A_{12}^{(1)}\right) A_{21}^{(2)} \\ g_1\left(A_{11}^{(2)} + A_{21}^{(2)}\right) A_{11}^{(1)} \\ g_1\left(A_{11}^{(2)} + A_{21}^{(2)}\right) A_{12}^{(1)} \end{pmatrix} \tag{9}$$

Here we define I_k and R as follows.

$$\begin{aligned} I_k &= A_{k1}^{(1)} + A_{k2}^{(1)} \\ R_k &= A_{1k}^{(2)} + A_{2k}^{(2)} \end{aligned} \tag{10}$$

From *eq.* (8), $\frac{d}{dt}(R_k - I_k) = 0$, and then $R_k - I_k = R_{k,0} - I_{k,0}$ (constant). From eq. (9), the following relation holds.

$$\frac{d}{dt}(A_{11}^{(2)} + A_{21}^{(2)}) \ge 2\eta g_1 (A_{11}^{(1)} + A_{12}^{(1)})(A_{11}^{(2)} + A_{21}^{(2)}) \tag{11}$$

$$\frac{d}{dt} R_k \ge 2\eta g_1 R_k \left(R_k - R_{k,0} + I_{k,0}\right). \tag{12}$$

Thus

$$\int_{R_0}^{R(t)} \{R_k\left(R_k - R_{k,0} + I_{k,0}\right)\}^{-1}\, dR_k \ge \int_0^t 2\eta g_1\, dt\,. \tag{13}$$

By solving this, we have

$$R_k(t) \ge \frac{R_{k.0}(R_{k.0} - I_{k.0})}{R_{k,0} - I_{k,0} \exp\left[2\eta g\left(R_{k,0} - I_0\right)t\right]}. \tag{14}$$

Supplementary Note 3. **Appearance time of bow-tie architecture.**

The lower bound of $R_k(t)$ diverges within the finite time, $t = \frac{1}{2\eta g}\frac{\ln R_{k,0} - \ln I_{k,0}}{R_{k.0} - I_{k,0}}$. For the situation of $R_0 \approx I_0$, from the definition of differentiation, the following equation is obtained:

$$t = \lim_{I_{k,0}\to R_{k.0}} \frac{1}{2\eta g}\frac{\ln R_{k,0} - \ln I_{k,0}}{R_{k,0} - I_{k,0}} = \frac{1}{2\eta g}\frac{d\left[\ln R_{k,0}\right]}{dR_{k,0}} = \frac{1}{2\eta g R_{k,0}}. \tag{15}$$

Thus, the column that has a larger $R_{k,0}$ diverges first. From this equation, R_k (t) is expected to diverge within a finite time. The divergence of $I_{k,0}$ and $R_{k,0}$ implies the emergence of bow-tie architecture (see Supplementary Fig. 9). Namely, in the early phase of evolution, bow-tie architecture emerges.

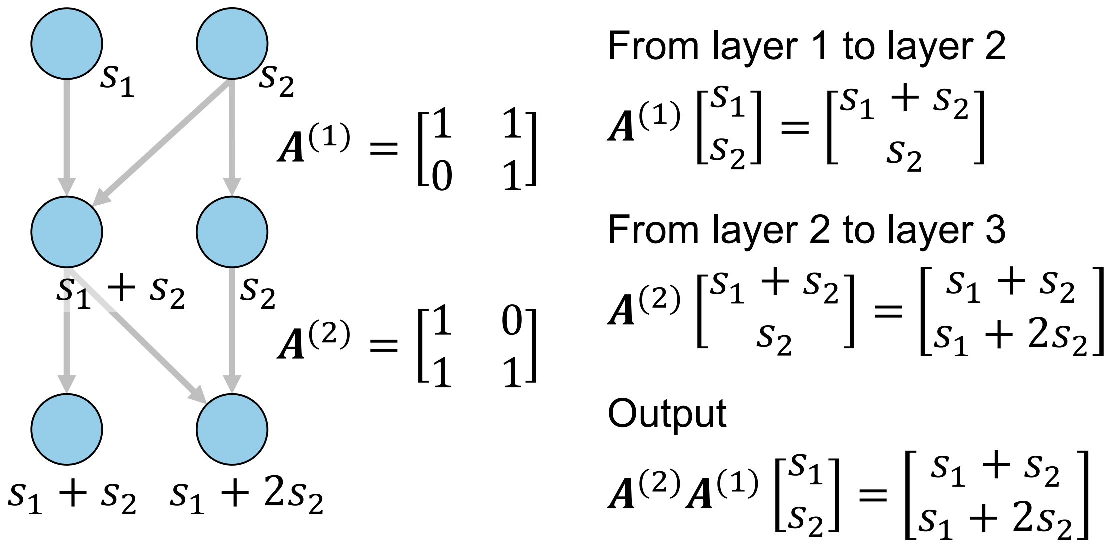

Supplementary Fig. 1. **Schematics of the linear network model with 2 nodes $\times$ 3 layers.** The activation level (i.e., expression level of the protein) is denoted near the node. The values are weighted by the link intensities and summed in the downstream node. In this network, the link intensities take a value of 0 or 1. The link intensity from node j in layer l to node i in layer $l+1$ is described by a $A_{ij}^{(l)}$. The matrices $\boldsymbol{A}^{(1)}$ and $\boldsymbol{A}^{(2)}$ next to the network describe the interactions between each pair of layers. The transmitted value between each pair of layers can be described by a product of matrices as shown on the right side of the figure.

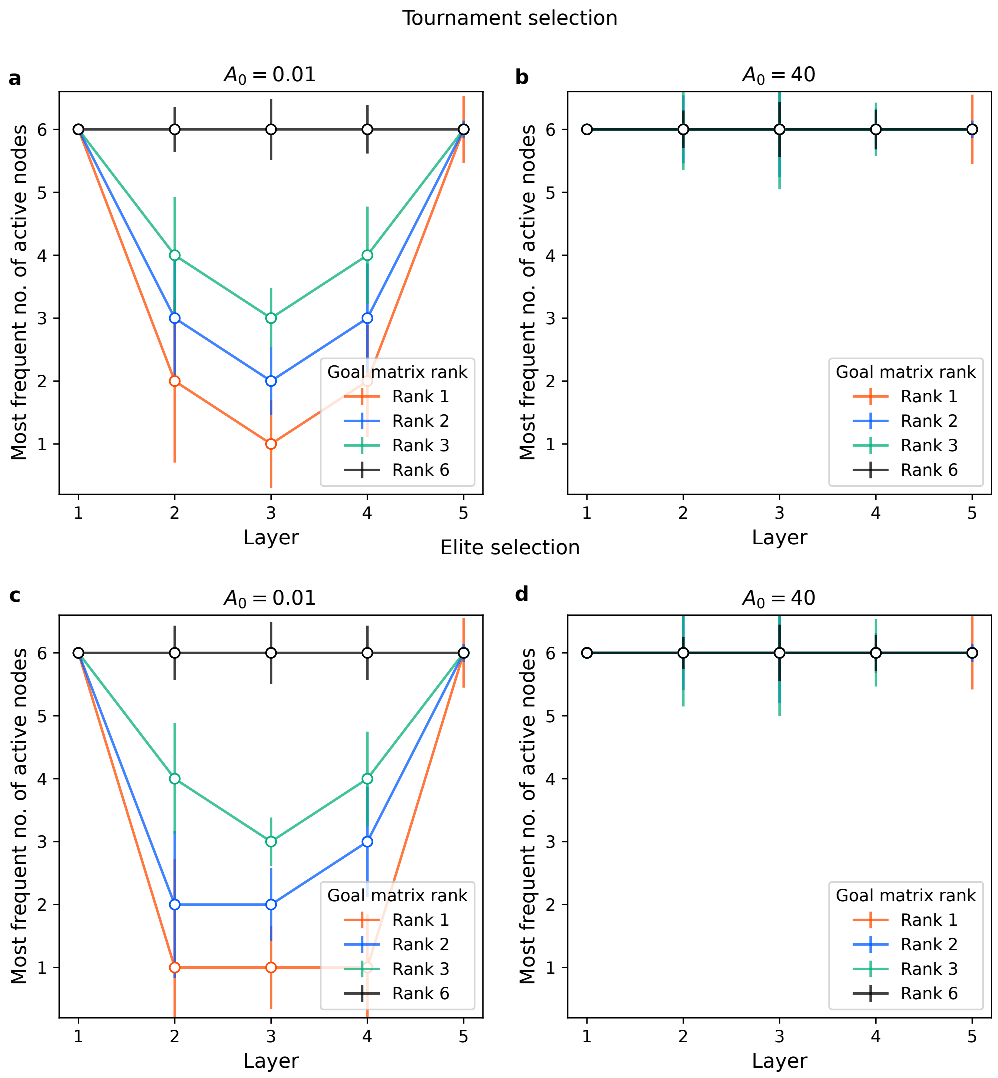

Supplementary Fig. 2. **Comparison of evolutionary simulation by tournament and elite selection for N=100.** Here, in GA with the elite selection, the top 25% of the population was selected in each generation. The Y-axis shows the mode among 100 runs of the number of active nodes in the most-adapted network at the end of the simulation. (**a, b**) Evolutionary simulation with tournament selection starting from (**a**) the small initial intensity and (**b**) the large initial intensity. (**c, d**) Evolutionary simulation with elite selection starting from (**c**) the small initial intensity and (**d**) the large initial intensity. The error bars are the standard deviation. The top left panel (**a**) is identical to Fig. 3A.

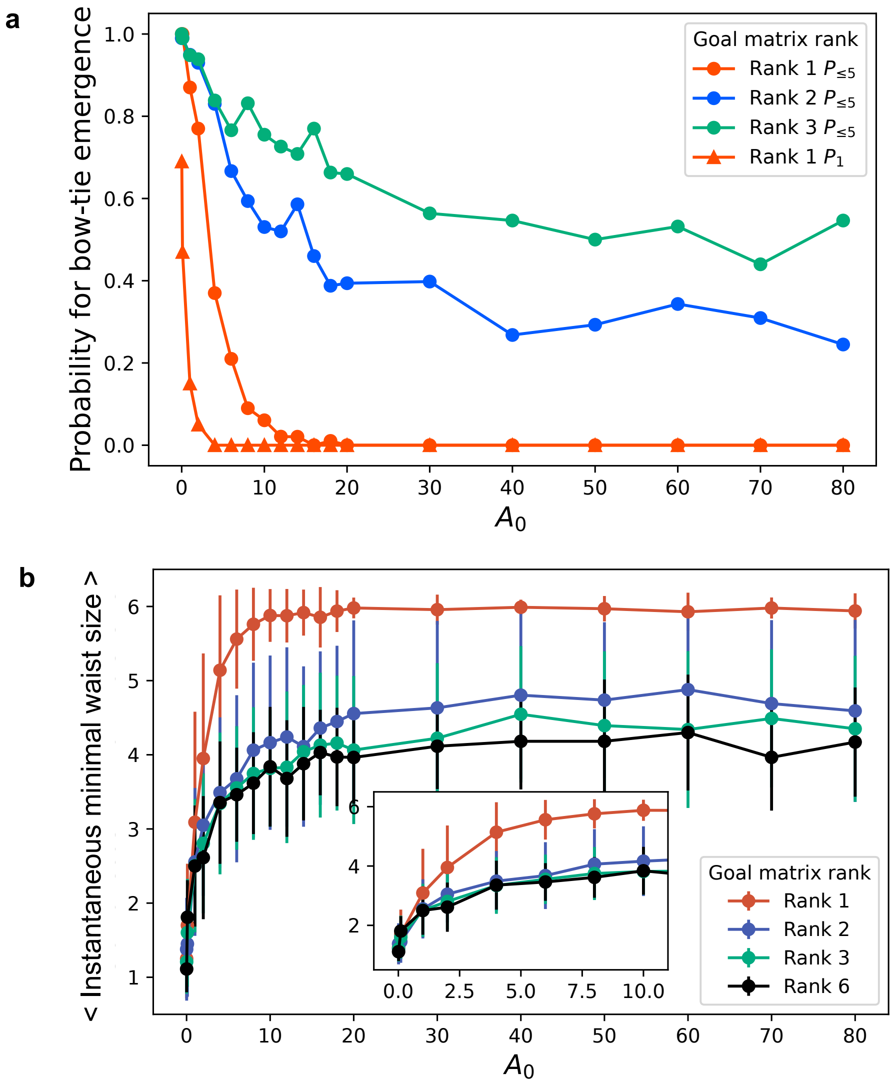

Supplementary Fig. 3. **Dependence of bow-tie emergence probability and instantaneous waist size on the initial value $\boldsymbol{A_0}$.** Evolutionary simulation started from various values of A_0. The X axis shows the link intensity of the initial network (A_0). Goal matrix elements are randomized under the conditions of rank 1 (red), rank 2 (blue), rank 3 (green), and rank 6 (black). The norm $\|\boldsymbol{G}\|_F$ is normalized to the same value ($\|\boldsymbol{G}\|_F = 60$). The simulation runs that reach F > -0.01 are used for the analysis. (**a**) Bow-tie emergence probability. The Y axis shows the probabilities of bowtie emergence P_1 and $P_{\leq 5}$, where P_1 denotes the probability that the number of active nodes in the 3rd layer (the waist) is 1, and $P_{\leq 5}$ represents the probability that the number of active nodes in the 3rd layer (the waist) is less than or equal to 5 (the sample size: n=100). Note that only results with the rank 1 goal are shown for P_1 and those with rank 1–3 goals are shown for $P_{\leq 5}$ since the probabilities for other ranks are almost constantly zero due to the fact that the waist size cannot be smaller than the goal rank in the steady state. (**b**) Instantaneous minimum waist size. The Y axis shows the instantaneous minimum waist size. Each dot represents the average among 100 runs. The inset is a magnification of results in the range A_0 = 0–10.

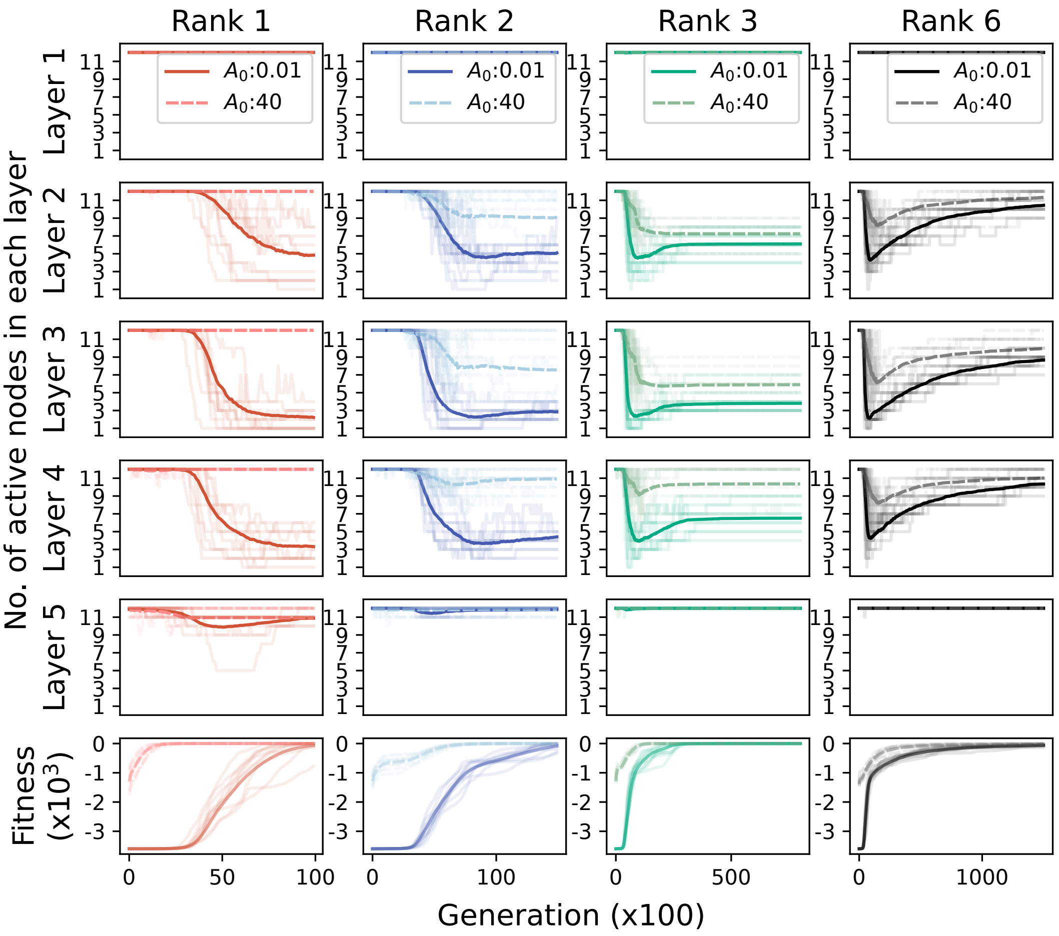

Supplementary Fig. 4. **Transient emergence of bow-tie architecture in the network with 12 nodes × 5 layers.** Evolutionary trajectories of the number of nodes in each layer for the network with $M = 12$. The bottom panel shows the average fitness trajectory. Simulation starts from $A_0 = 0.01$ in the solid lines and $A_0 = 40$ in the dashed lines. Trajectories are averaged among independent simulation runs (*n=100* for each color). The trajectories of 10 independent runs are shown with a paler shade. Goal matrix elements are randomized under the conditions of rank 1 (red), rank 2 (blue), rank 3 (green), and rank 6 (black). The norm $\|\boldsymbol{G}\|_F$ is normalized to the same value ($\|\boldsymbol{G}\|_F = 60$). The simulation runs that reach $F > -0.01$ are used for the analysis.

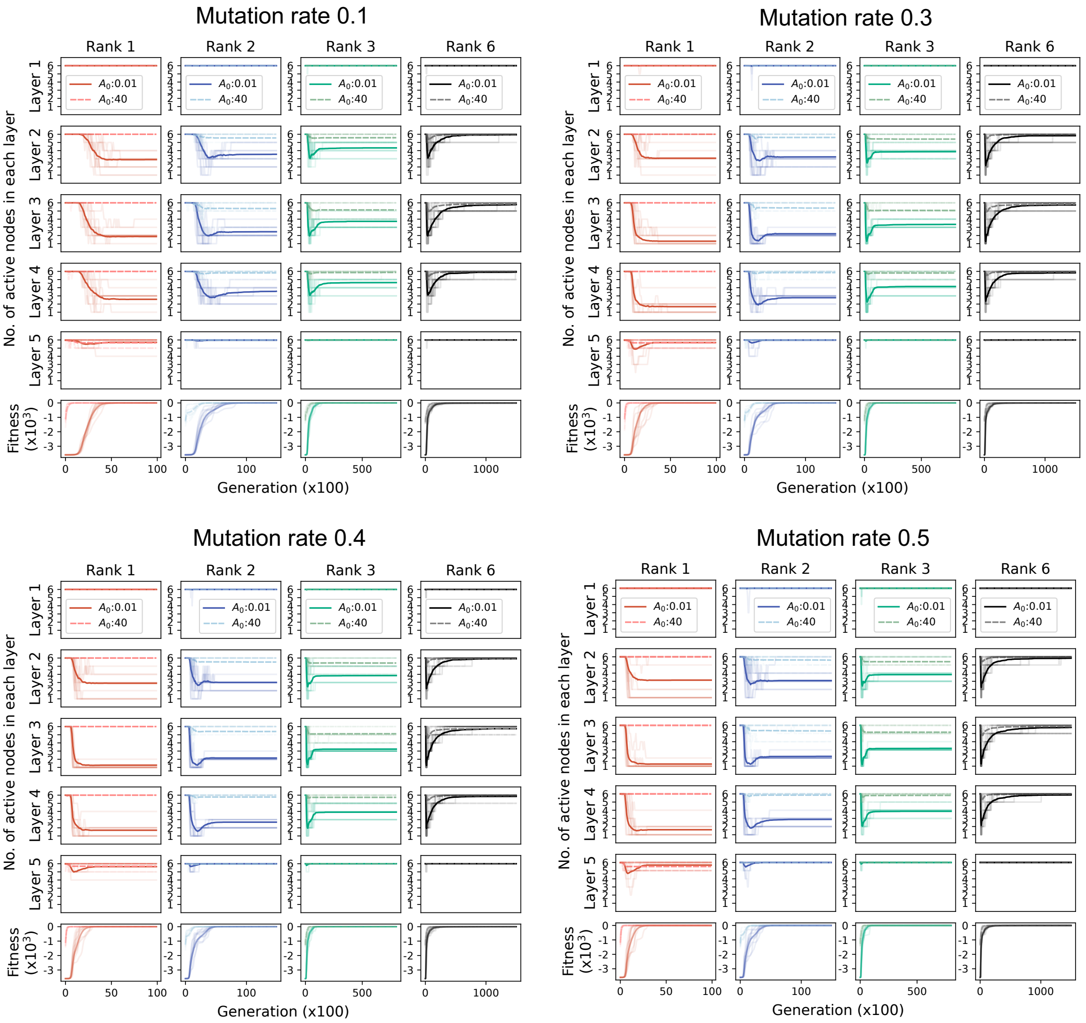

Supplementary Fig. 5. **Transient emergence of bow-tie architecture in the various mutation rates.** Evolutionary trajectories of the number of nodes in each layer for the network with $M = 6$. The bottom panel shows the average fitness trajectory. Simulation starts from $A_0 = 0.01$ in the solid lines and $A_0 = 40$ in the dashed lines. Trajectories are averaged among independent simulation runs (*n=100* for each color). The trajectories of 10 independent runs are shown with a paler shade. Goal matrix elements are randomized under the conditions of rank 1 (red), rank 2 (blue), rank 3 (green), and rank 6 (black). The norm $\|\boldsymbol{G}\|_{\mathrm{F}}$ is normalized to the same value ($\|\boldsymbol{G}\|_{\mathrm{F}} = 60$). The simulation runs that reach $F > -0.01$ are used for the analysis. Bow-tie architecture transiently emerges under the mutation rates 0.1, 0.3, 0.4 and 0.5, which is the same result as for the mutation rate 0.2 used in the main text.

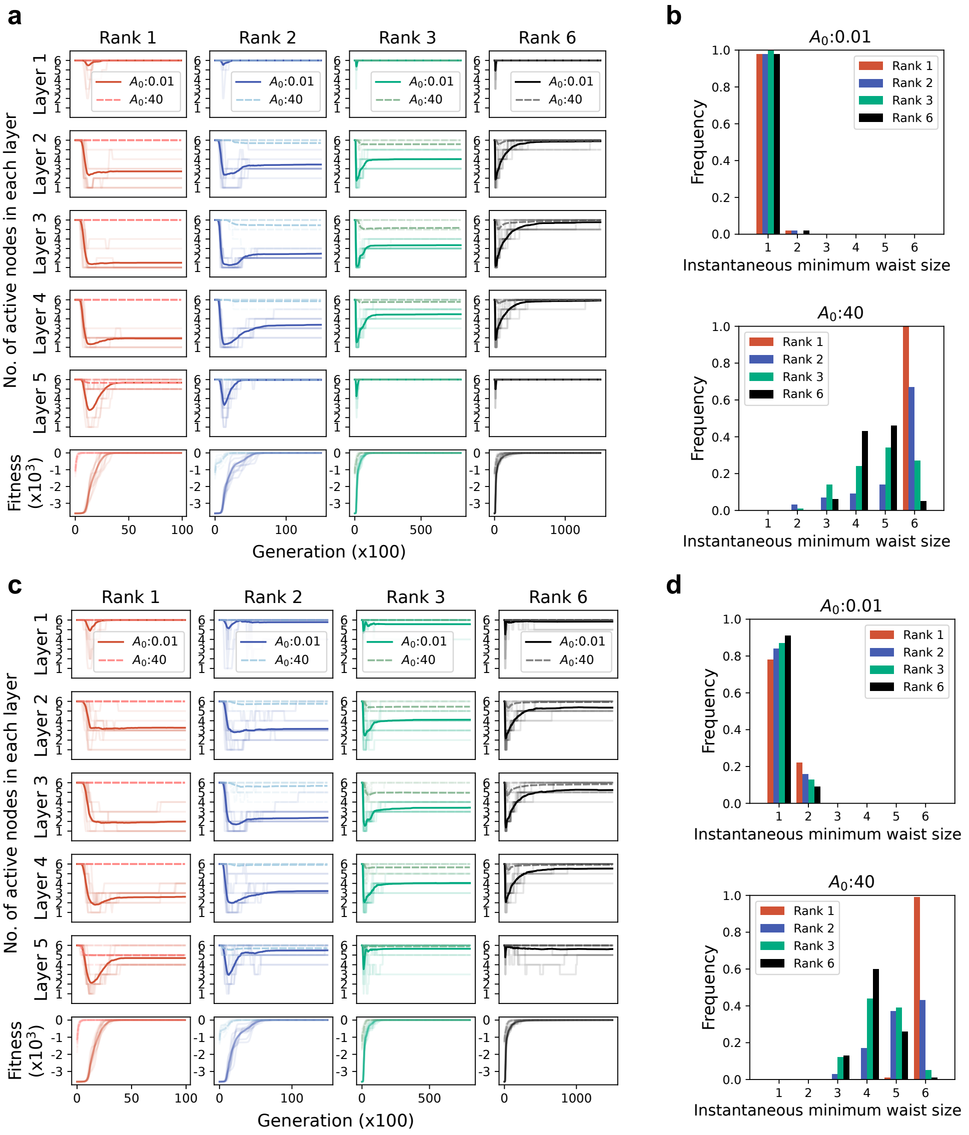

Supplementary Fig. 6. **Evolutionary trajectories of the number of nodes in alternative definitions of active nodes.** (**a**) Evolutionary trajectories of the number of nodes in each layer and (**b**) the distribution of the instantaneous minimum waist size (sample size: n=100 for each color) for the case where the active node is defined as the relative contribution to the total in-out relation (see Methods). (**c**) Evolutionary trajectories and (**d**) the distribution of the instantaneous minimum waist size (sample size: n=100 for each color) for the case where the active node is defined as the relative strength of maximum interactions (see Methods). In the trajectories (**a, c**), simulation starts from $A_0 = 0.01$ in the solid lines and $A_0 = 40$ in the dashed lines. Trajectories are averaged among independent simulation runs (n=100 for each color). The trajectories of 10 independent runs are shown with a paler shade. The bottom row in panels (**a**) and (**c**) shows the average fitness trajectory. For each simulation, the goal matrix elements are randomized under the conditions of rank 1 (red), rank 2 (blue), rank 3 (green), and rank 6 (black). The norm $\|\boldsymbol{G}\|_{\mathrm{F}}$ is normalized to the same value ($\|\boldsymbol{G}\|_{\mathrm{F}}$ = 60). The simulation runs that reach F > -0.01 are used for the analysis.

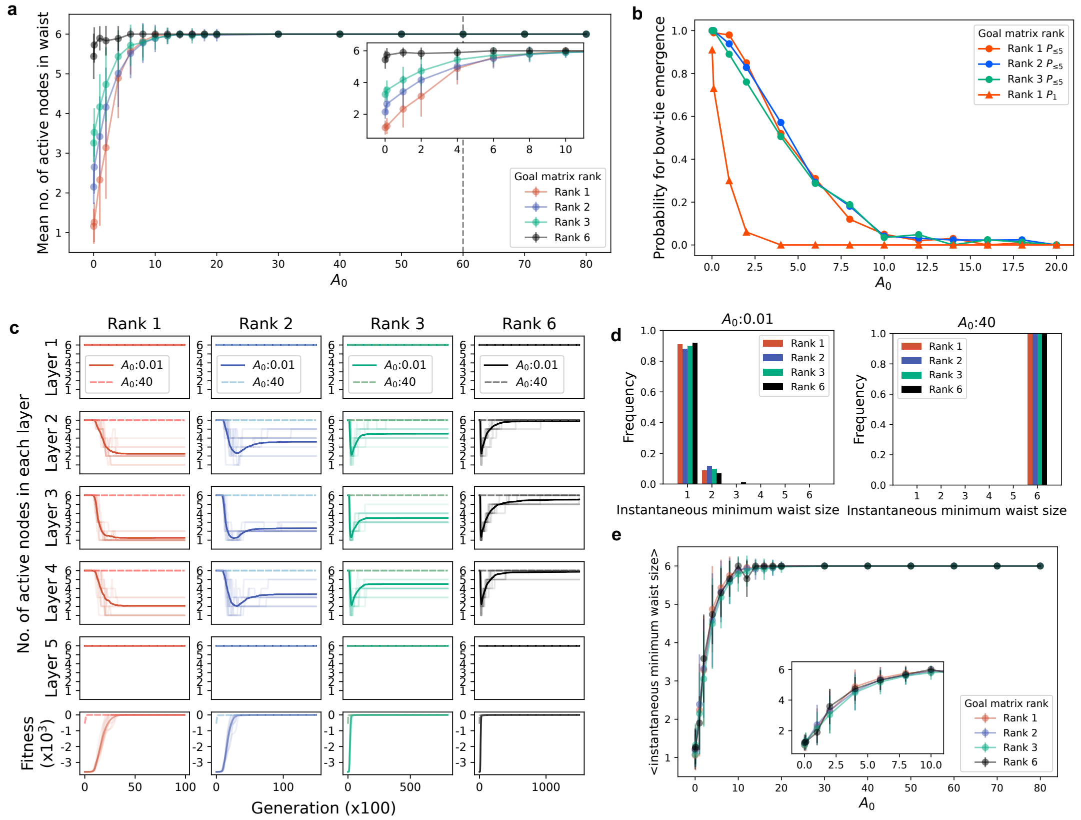

Supplementary Fig. 7. **Evolutionary simulation where the variance in goal matrix elements is normalized.** (**a**) The dependence of bow-tie emergence on the initial link intensity for the goal matrix in which the variance in goal matrix elements is normalized. X-axis: The link intensity of the initial network. Y-axis: Mean number of active nodes in the waist layer in an adapted network among 100 runs. The error bars represent the standard deviation. The inset is a magnification of results in the range $A_0 =$ 0–10. (**b**) The probabilities of bowtie emergence when the variance in goal matrix elements is normalized. P_1 denotes the probability that the number of active nodes in the 3rd layer (the waist) is 1, and $P_{\leq 5}$ represents the probability that the number of active nodes in the 3rd layer (the waist) is less than or equal to 5. (**c**) Evolution trajectories of the number of nodes in each layer for the variance normalized goal. The bottom panel shows the average fitness trajectory. Simulation starts from $A_0 = 0.01$ in the solid lines and $A_0 = 40$ in the dashed lines. Trajectories are averaged among independent simulation runs (n=100 for each color). The trajectories of 10 independent runs are shown with a paler shade. (**d**) Distribution of the instantaneous minimum waist size that the network experienced during evolution (n=100 for each color). Evolution started from a small value ($A_0 = 0.01$) on the left and a large value on the right ($A_0 = 40$). (**e**) The initial value dependence of the instantaneous minimum waist size for the variance-normalized goal matrix. The X axis shows the link intensity of the initial network (A_0). The Y axis shows the instantaneous minimum waist size. Each dot is an average among 100 runs. The inset is a magnification of results in the range $A_0 = 0 - 10$. Goal matrix elements are randomized under the conditions of rank 1 (red), rank 2 (blue), rank 3 (green), and rank 6 (black). The norm $\|\boldsymbol{G}\|_{\mathrm{F}}$ and variance of goal matrix elements $V_{G_{ij}}$ are normalized to the same value respectively ($\|\boldsymbol{G}\|_{\mathrm{F}} = 60$; $V_{G_{ij}} = 1$; see also Methods). The simulation runs that reach $F >$ -0.01 are used for the analysis.

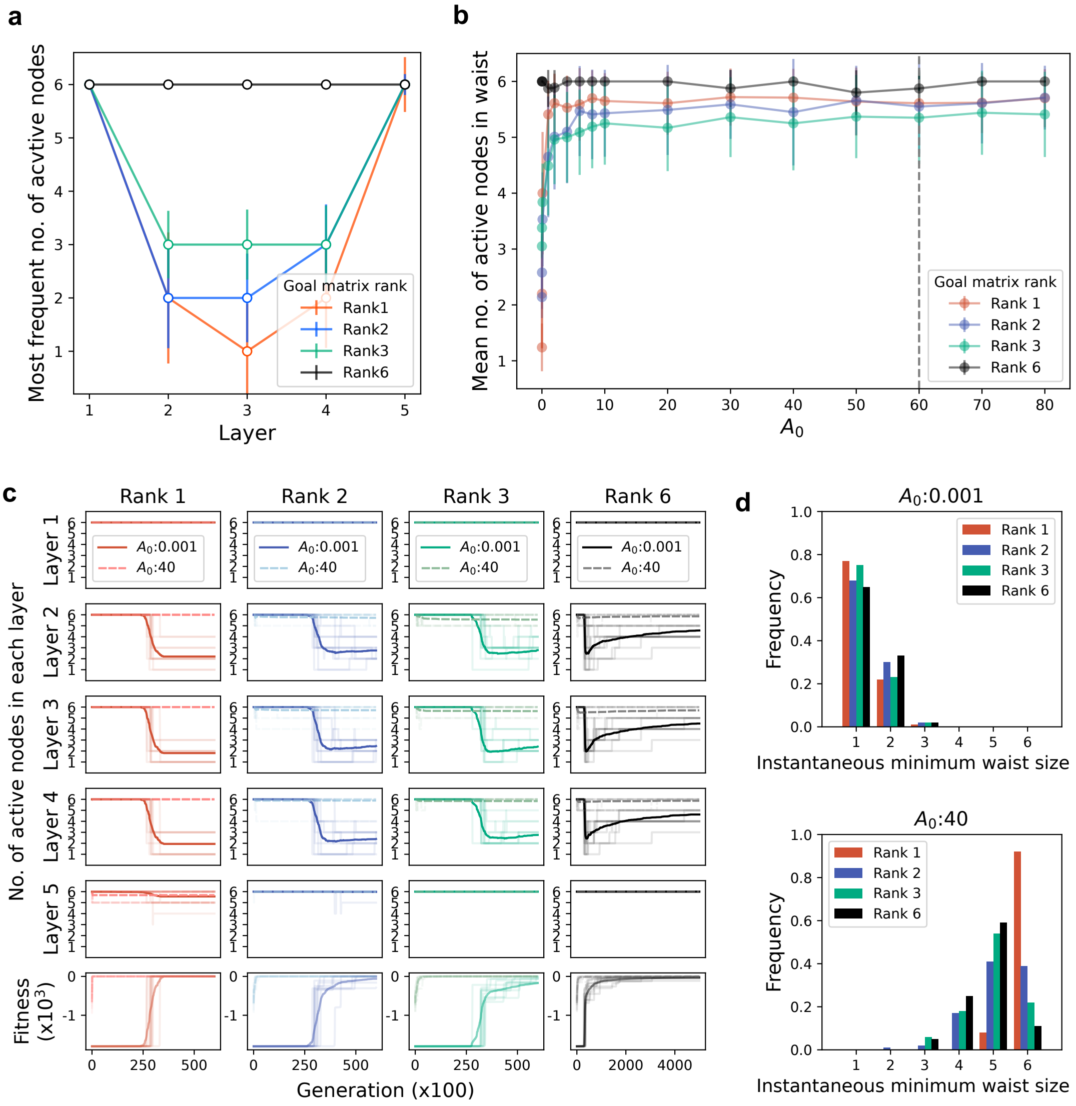

Supplementary Fig. 8. **Simulation results for a network with 6 nodes x 5 layers in the ODE model.** The numerical integration is performed by the fourth-order Runge–Kutta method. (**a**) The mode among 100 runs of the number of active nodes in the most-adapted network. The number of runs is [rank 1: 100; rank 2: 100; rank 3: 99; rank 6: 5]. The initial link intensity is set to A_0: 0.01. The error bars represent the standard deviation. Goal matrix elements are randomized under the conditions of rank 1 (red), rank 2 (blue), rank 3 (green), and rank 6 (black). The norm $\|\boldsymbol{G}\|_\mathrm{F}$ is normalized to the same value ($\|\boldsymbol{G}\|_\mathrm{F} = 60$). (**b**) The bow-tie emergence exhibits a strong dependence on the initial link intensity. X-axis: The link intensity of the initial network. Y-axis: Mean number of active nodes in the waist layer in an adapted network among 100 runs. The error bars are the standard deviation. The dashed line shows $\|\boldsymbol{G}\|_\mathrm{F} = 60$. (**c**) Evolution trajectories of the number of nodes in each layer. Simulation starts from $A_0 = 0.001$ in the solid lines and $A_0 = 40$ in the dashed lines. Trajectories are averaged among independent simulation runs (n=100 for each color). The simulation runs that reach F > -0.01 are used. (**d**) Distribution of instantaneous minimum waist size that the network experienced during evolution (n=100 for each color).

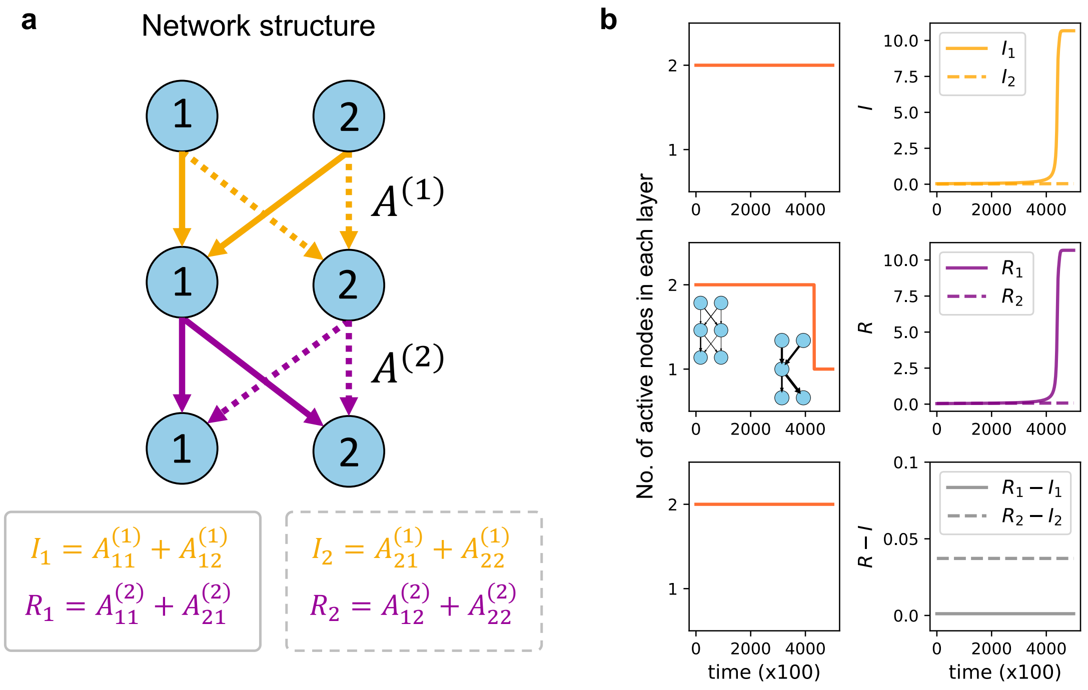

Supplementary Fig. 9. **Schematic illustration of the mathematical analysis.** (**a**) A 2×3 layer network described by 2 matrices $\boldsymbol{A}^{(1)}$ and $\boldsymbol{A}^{(2)}$. The links denoted by the thick lines (I_1: yellow; R_1: purple) are evolved independently of the dashed lines (I_2: yellow; R_2: purple). Each pair of I_i and R_i is interdependent by the constraint of R_i–I_i= const when $\|\boldsymbol{A}\|_{\mathrm{F}} \ll \|\boldsymbol{G}\|_{\mathrm{F}}$. (**b**) Evolution toward the rank 1 goal matrix ($\|\boldsymbol{G}\|_{\mathrm{F}} = 60$) from the initial norm $A_0 =$ 0.01. Left panel: Evolutionary trajectories of the network. Y-axis: Number of nodes in each layer. The initial norm A_0 is 0.01. Right panel: Evolutionary trajectory of *I*, *R*, and *R*-*I*. Solid lines show R_1, I_1 and R_1- I_1 and dashed lines show R_2, I_2 and R_2- I_2. The divergence of R_1 and I_1 is accompanied with the emergence of the narrow waist in the network.

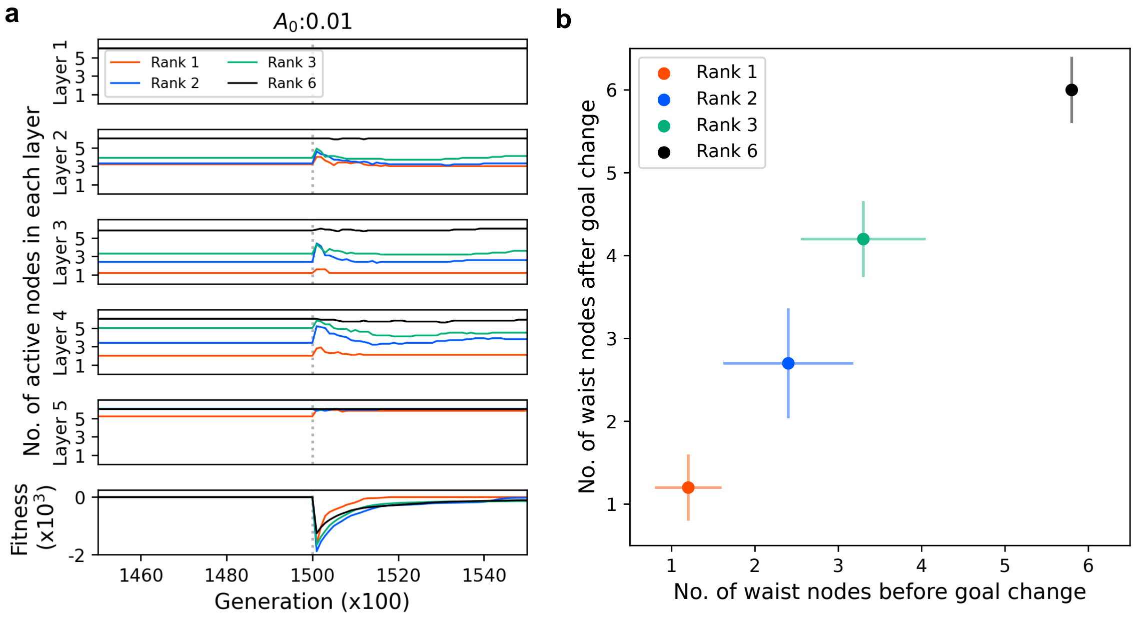

Supplementary Fig. 10. **Bow-tie architecture is maintained after changing the goal matrix.** (**a**) Evolutionary trajectories of the network from the initial norm $A_0 = 0.01$ for the goal matrix with rank 1 (red), rank 2 (blue), rank 3 (green), and rank 6 (black). The goal matrix is altered at the 150,000th generation (dashed line). Trajectories are averaged among independent simulation runs (n=10). X-axis: Generation. Y-axis: Number of nodes in each layer. (**b**) Comparison between the first and second adapted networks. X-axis: Waist size of the network at the 150,000th generation (i.e., before the goal change). Y-axis: Waist size of the network at the 300,000th generation (i.e., after the goal change). Each dot is the average among 10 runs, and the error bars are the standard deviation.

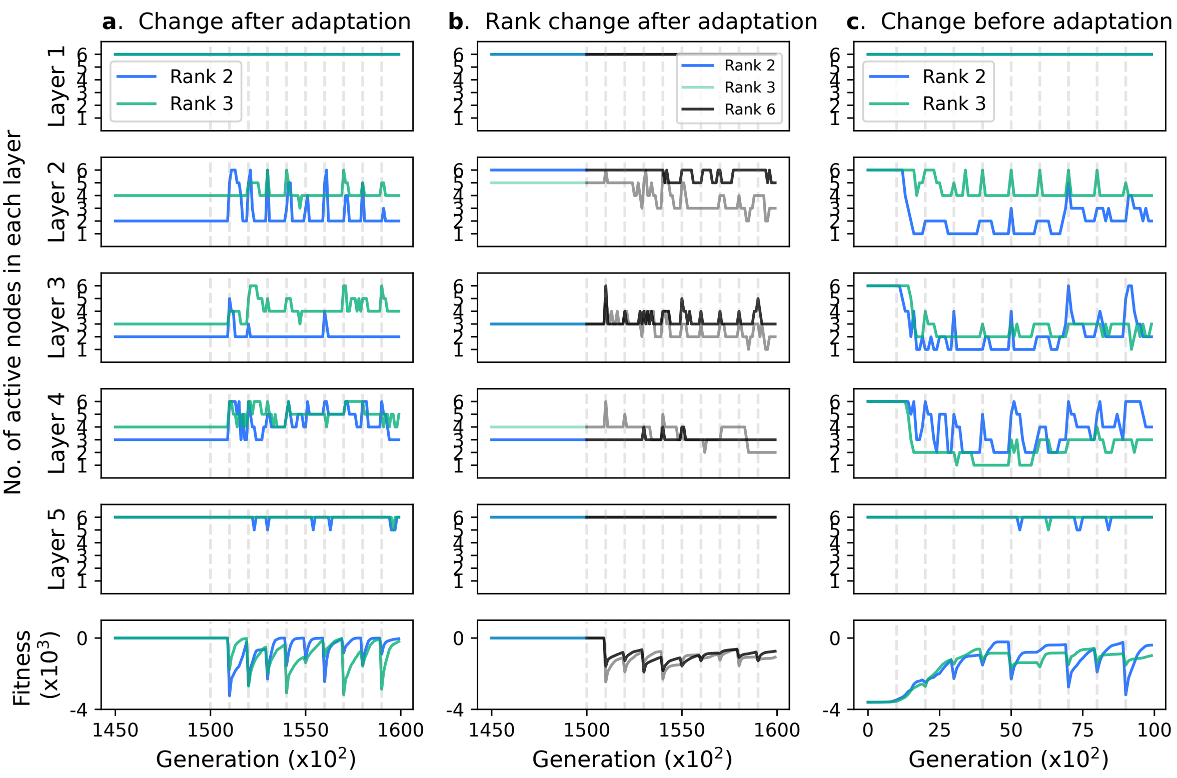

Supplementary Fig. 11. **Introduction of goal matrix fluctuation in a network with *M=6 L=4* (5 layers) when the goal matrix is rank 2 and rank 3.** The goal matrix is changed every 1,000 generations (dashed line) after or before the adaptation. The number of nodes in each layer (upper 5 panels) and the fitness of the most-adapted network in the population (the bottom panel) are plotted against the generation. (**a**) Sequential goal matrix changes after fitness is converged. The goal matrix changes without changing the norm and rank (blue: rank 2; green: rank 3). (**b**) Sequential goal matrix changes after fitness is converged. The goal matrix rank is changed from rank 2 to rank 6 (dark color), or from rank 3 to rank 6 (pale color) in the first change. (**c**) Sequential goal matrix changes from the beginning of evolution. The goal matrix rank and norm do not change (blue: rank 2; green: rank 3).

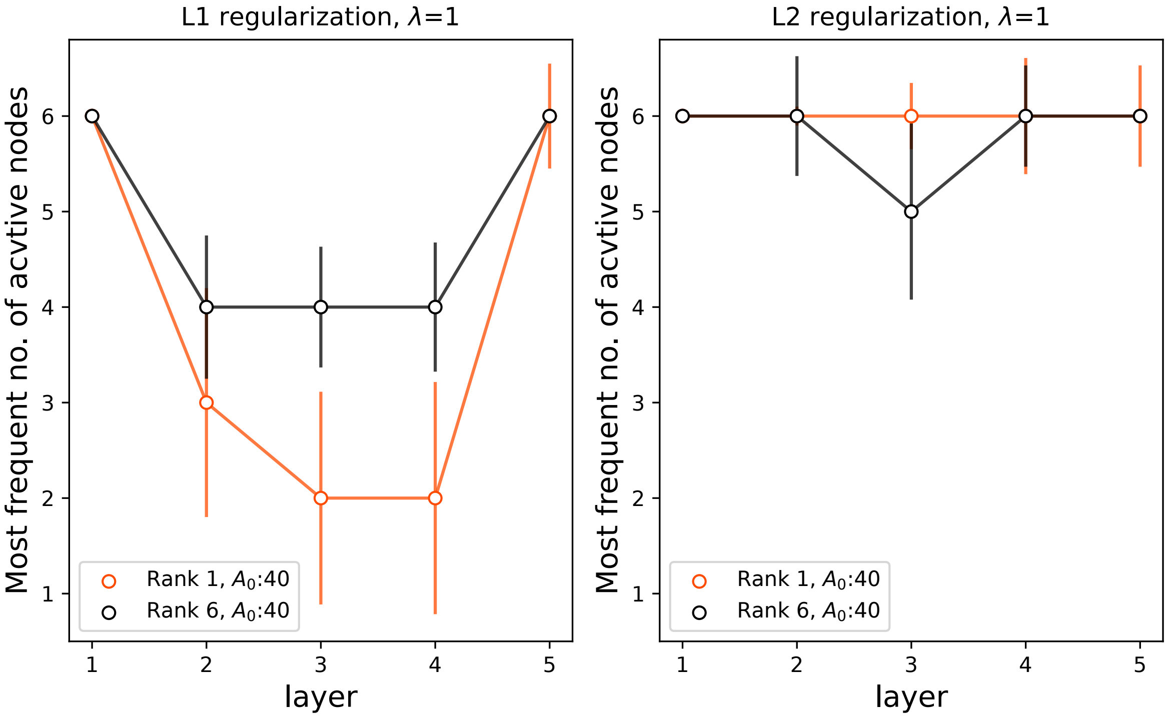

Supplementary Fig. 12. **Bow-tie evolution under the L1 or L2 regularization term.** Evolutionary simulation for a bow-tie architecture including the L1 (left) or L2 (right) regularization term as the cost for maintaining link intensities. The Y-axis shows the mode among 100 runs of the number of active nodes in the most-adapted network. The number of runs is [rank 1: 100; rank 6: 100]. Goal matrix elements are randomized under the conditions of rank 1 (red) and rank 6 (black). Initial link intensities are set to $A_0 = 40$. The regularization coefficient λ =1 is used. The error bars represent the standard deviation.

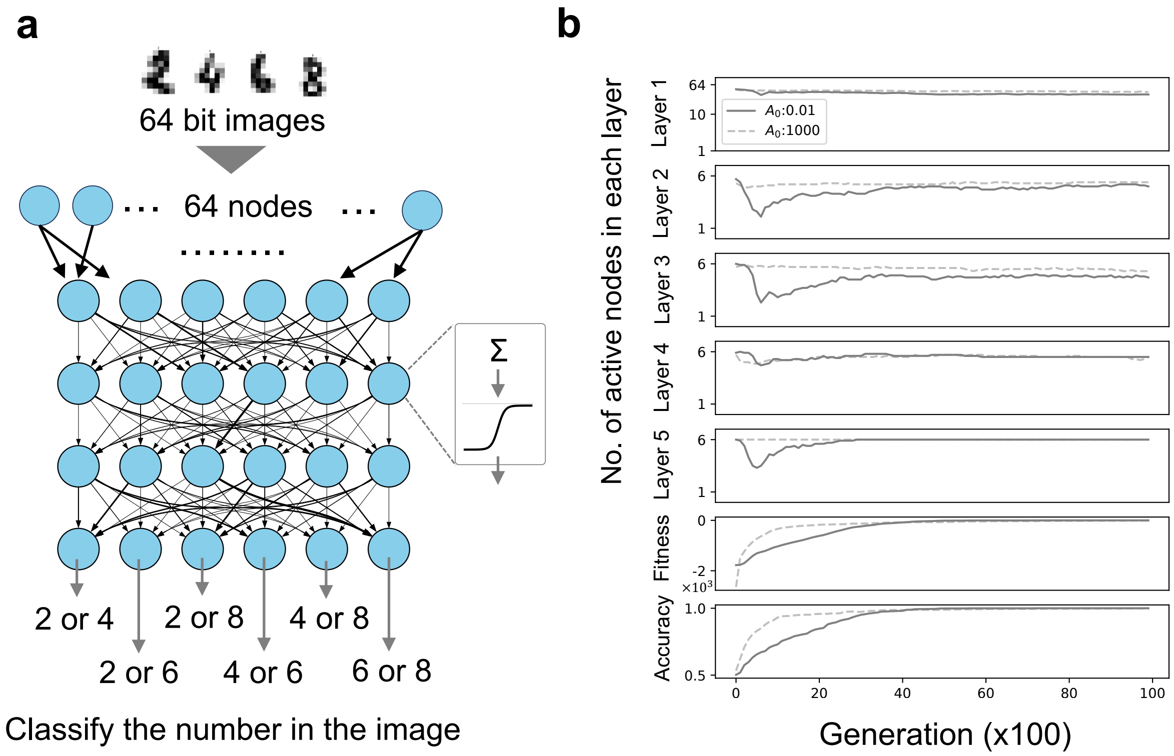

Supplementary Fig. 13. **Emergence of bow-tie architecture in a non-linear network.** (**a**) Non-linear network is evolved to classify the 64 digit image into the redundant 6 groups that consist of (2 or 4), (2 or 6), (2 or 8), (4 or 6), (4 or 8), and (6 or 8). The network receives images (64-length array) of handwritten numbers ("2", "4", "6" or "8"), and outputs a prediction of the groups to which the written number belongs with a 6-bit array. (**b**) Evolutionary trajectories of the network. X-axis represents generation. The upper 5 panels show the mean number of active nodes in each layer among 10 simulation runs. The second panel from the bottom shows mean fitness that is defined as the distance between a current output and an ideal output. The bottom panel shows the accuracy score which represents how precisely the network can classify the images. Evolution starts from a large value (A_0: 100) in the dashed line and from a small value in the solid line (A_0: 0.01).
